# Immuno‐Nanocomplexes Target Heterogenous Network of Inflammation and Immunity in Myocardial Infarction

**DOI:** 10.1002/advs.202402267

**Published:** 2024-07-25

**Authors:** Fan Su, Weifan Ye, Yi Shen, Yujie Xie, Chong Zhang, Qianyun Zhang, Zhengqi Tang, Meihua Yu, Yu Chen, Bin He

**Affiliations:** ^1^ Department of Critical Care Medicine Shanghai Chest Hospital, Shanghai Jiao Tong University Shanghai 200030 P. R. China; ^2^ Materdicine Lab, School of Life Sciences Shanghai University Shanghai 200444 P. R. China; ^3^ Shanghai Institute of Materdicine Shanghai 200051 P. R. China

**Keywords:** immunotherapy, myocardial infarction, nanoemulsion, neutrophilic inflammation, ROS scavenger

## Abstract

Despite the proceeds in the management of acute myocardial infarction (AMI), the current therapeutic landscape still suffers from limited success in the clinic. Exaggerated inflammatory immune response and excessive oxidative stress are key pathological features aggravating myocardium damage. Herein, catalytic immunomodulatory nanocomplexes as anti‐AMI therapeutics to resolve reactive oxygen species (ROS)‐proinflammatory neutrophils‐specific‐inflammation is engineered. The nanocomplexes contain lyophilic S100A8/9 inhibitor ABR2575 in the core of nanoemulsions, which effectively disrupts the neutrophils‐S100A8/A9‐inflammation signaling pathway in the AMI microenvironment. Additionally, ROS scavenger ultrasmall Cu_x_O nanoparticles are incorporated into the nanoemulsions via coordinating with SH groups of poly(ethylene glycol) (PEG)‐conjugated lipids, which mimic multiple enzymes, dramatically alleviating the oxidative stress damage to myocardial tissue. This combination strategy significantly suppresses the infiltration of pro‐inflammatory monocytes, macrophages, and neutrophils, as well as the secretion of inflammatory cytokines. Additionally, it potentially triggers cardiac Tert activation, which promotes myocardial function and decreases infarction size in preclinical murine AMI models. This approach offers a new nanomedicine for treating AMI, resulting in a dramatically enhanced therapeutic outcome.

## Graphical abstract

This study constructs a catalytic immunomodulatory nanoemulsion for effective treatment of acute myocardial infarction (AMI) by targeting ROS‐neutrophilic inflammation. This approach effectively disrupts neutrophil‐S100A8/A9 signaling pathway, alleviates the levels of multiple radicals, and activates cardiac Tert signaling pathway, thereby remarkably enhancing myocardial functions, and reducing infarction sizes of AMI mice.

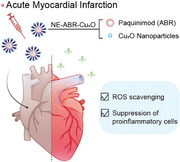

## 1 Introduction

AMI remains one of the leading causes of death and frailty in the modern world, despite significant progress in the field of AMI management.^[^ ^1^ ^]^ About 40% of AMI patients still have long‐term or even life‐lasting heart failure.^[^ ^2^ ^]^ Myocardial infarction triggers the infiltration of massive heterogeneous subsets of pro‐inflammatory immune cells, of which the frequency of infiltrating neutrophils is positively associated with increased infarct size and declined heart function, representing an independent prognostic factor in AMI patients.^[^ ^3^ ^]^ Therefore, a broad spectrum of treatments has been explored to target neutrophils in animal AMI models, including neutrophils‐derived alarmins S100A8/A9 inhibitors (ABR2575),^[^ ^4^ ^]^ metoprolol (β1‐adrenergic‐receptor antagonist),^[^ ^5^ ^]^ and anti‐Ly6G antibody (neutrophil depletion).^[^ ^6^ ^]^ Although the significant improvements of these strategies in cardiac function and tissue injury are evident, their clinical translation has yet to be unsuccessful. This is mainly caused by the inappropriate pharmacokinetic distribution of these small molecular drugs with suboptimal effectiveness and unsatisfied safety profiles.^[^ ^7^ ^]^ Emerging advanced nanoplatforms with finely tuned physiochemical features can markedly alter pharmacokinetics, biodistribution, and targeted delivery to diseased tissue.^[^ ^8^ ^]^ The implementation of nano‐engineering means to target AMI‐specific inflammatory immune cells has rarely been reported.^[^ ^9^ ^]^

Massively increased ROS immediately post‐myocardial infarction is another key pathological feature in the AMI microenvironment, which in turn enhances the infiltration of circulating immune cells, the activation of pro‐inflammatory phenotype (M1‐type) macrophages, and the production of pro‐inflammatory cytokines.^[^ ^10^ ^]^ Excessive ROS further deteriorates the inflammatory microenvironment and damages the myocardial cells.^[^ ^11^ ^]^ Meanwhile, the intensive level of ROS also greatly diminishes the therapeutic effects of small drugs. Therefore, eliminating ROS to suppress oxidative stress injury and improve drug efficacy is critically important for AMI treatment.

Beyond transport function, certain types of nanoparticles intrinsically possess ROS‐scavenging capability and have demonstrated their potential in the treatment of distinct inflammatory diseases, including nanoenzymes for AMI treatment,^[^ ^12^ ^]^ ultrasmall metal nanoparticles (e.g., Cu_5.4_O) for treating acute lung/kidney/liver injury,^[^ ^13^ ^]^ 2D MXene as therapeutics for the treatment of neurodegenerative diseases and hypertension,^[^ ^14^ ^]^ black phosphorus nanosheets for osteoarthritis treatment,^[^ ^15^ ^]^ carbon dots for ischemic stroke treatment,^[^ ^16^ ^]^ and ceria nanoclusters for depression treatment.^[^ ^17^ ^]^ However, the integration of nano‐ROS scavenger with neutrophil‐targeting therapy has been scarcely exploited for enhanced AMI immunotherapy.

**Scheme 1:**
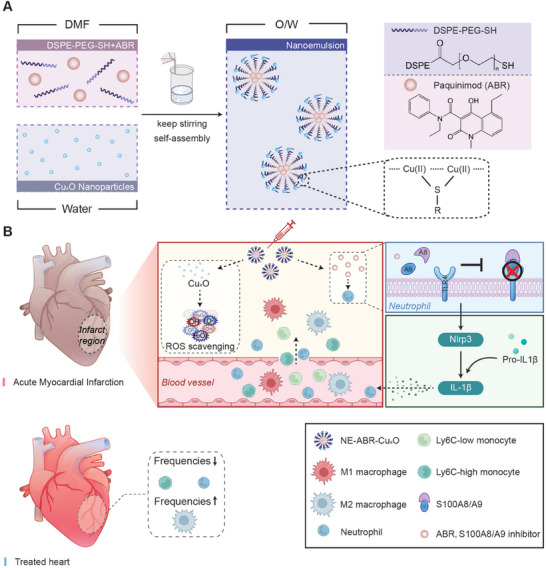
An illustrative scheme shows A) the construction of NE‐ABR‐Cu_x_O nanocomplexes via a nanoemulsion method, and B) the mechanism of action in alleviating the oxidative stress and regulating neutrophilic inflammation for effective treatment of acute myocardial infarction.

In the present study, we developed catalytic immunomodulatory nanoemulsion complexes for the treatment of AMI using a self‐assembly agent, 1,2‐distearoyl‐sn‐glycero‐3‐phosphoethanolamine conjugated PEG‐thiol (DSPE‐PEG‐SH), where S100A8/A9 inhibitor ABR molecules were encapsulated in the lyophilic core and Cu_x_O nanoparticles were coordinated with thiol groups on the surface of nanoemulsions (NE‐ABR‐Cu_x_O, **Scheme** **1**A). Upon arriving at the infracted area (Scheme 1B), Cu_x_O nanoparticles effectively scavenged distinct types of ROS, alleviating oxidative stress. Additionally, ABR blocked the activation of exaggerated neutrophils infiltrated in the infarct area by interrupting the S100A8/A9‐NLPR3‐IL‐1β inflammation signaling pathway, thereby suppressing inflammatory immune responses. In particular, the frequencies of pro‐inflammatory phenotypes of innate immune cells, such as Ly6C‐high monocytes and neutrophils, dramatically decreased in the damaged myocardial tissue, while anti‐inflammatory phenotypic M2 macrophages were significantly increased. Beyond that, RNA sequencing analysis uncovered that NE‐ABR‐Cu_x_O enhanced cardioprotection was also potentially medicated by a new therapeutic pathway of cardiac Tert activation. NE‐ABR‐Cu_x_O nanocomplexes combination therapy markedly repaired the myocardial function and decreased the infarction size with a long‐term protection effect against murine AMI, offering great potential in clinical invention implementation.

## 2 Results and Discussion

### 2.1 Construction and Characterization of NE‐ABR‐Cu_x_O

**Figure 1:**
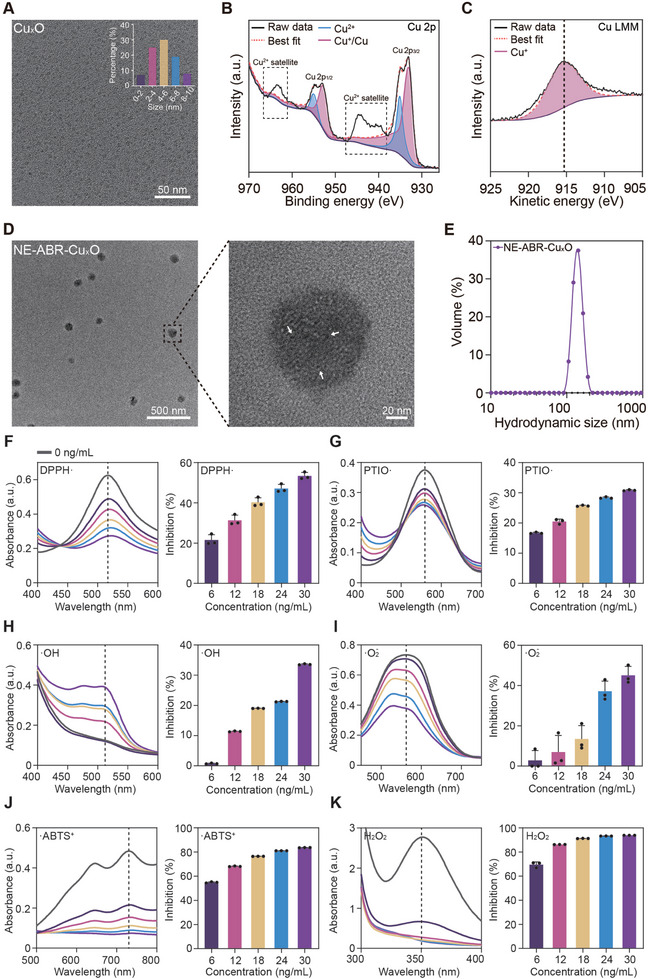
Characterization and radical‐scavenging capability of NE‐ABR‐Cu_x_O. A) A TEM image of Cu_x_O nanoparticles and their size distribution by measuring 100 nanoparticles (inset). B) Cu 2p XPS and C) Cu LMM spectra of Cu_x_O nanoparticles. D) TEM images of NE‐ABR‐Cu_x_O. E) The hydrodynamic diameter distribution curve of NE‐ABR‐Cu_x_O in PBS (phosphate‐buffered saline) solution by DLS measurement. F–K) UV−vis spectra of detection agents and quantitative analysis of free‐radical scavenging ability of NE‐ABR‐Cu_x_O toward F) DPPH·, G) PTIO·, H) ·OH, I) ·O‐ 2, J) ·ABTS^+^, and (K) H_2_O_2_. All data are shown as the mean ± standard deviation (SD).

To prepare NE‐ABR‐Cu_x_O complexes, Cu_x_O ultrasmall nanoparticles were first obtained by a reduction reaction between CuCl_2_ and L‐ascorbic acid, which were suspended in an aqueous solution. The dimethylformamide (DMF) oil phase that contained DSPE‐PEG‐SH monomer and ABR inhibitor molecules was slowly mixed with the aqueous phase to form nanoemulsions, where the Cu_x_O nanoparticles were coordinated with SH groups of monomers. The resultant NE‐ABR‐Cu_x_O complexes were harvested via dialysis after the removal of DMF. Transmission electron microscopy (TEM) images revealed that Cu_x_O nanoparticles displayed an ultrasmall size of 5 nm (**Figure** **1**A). X‐ray photoelectron spectroscopy (XPS) was performed to assess the chemical valence states of Cu in Cu_x_O. The survey spectrum clearly implied the characteristic peaks of Cu 2p_1/2_ at 953.0 eV and Cu 2p_3/2_ at 933.0 eV (Figure S1, Supporting Information). Further analysis of the XPS spectrum of Cu 2p revealed strong characteristic peaks of Cu/Cu^+^ at 952.9 and 933.2 eV (Figure 1B).^[^ ^18^ ^]^ The typical peaks at 955.0 eV and 935.2 eV and their satellite peaks at 963.0 and 943.0 eV suggest a significant proportion of Cu^2+^. To differentiate Cu and Cu^+^, the Cu LMM (L‐inner level‐M‐inner level‐M‐inner level electron transition) Auger spectrum was collected. The results showed an Auger electron kinetic energy peak at 915.4 eV, which corresponded to the typical peak of Cu^+^ (Figure 1C; Figure S1, Supporting Information). Collectively, Cu_x_O exhibited the coexistence of two copper species of Cu^2+^ and Cu^+^. Following assembly with ABR, the obtained NE‐ABR‐Cu_x_O complexes were well dispersed and spherical in morphology with a size of ≈100 nm (Figure 1D, left). the Cu_x_O nanoparticles were clearly observed in the spheres, as indicated by the white arrows (Figure 1D, right). Dynamic light scattering (DLS) analysis suggested that the average hydrodynamic size of NE‐ABR‐Cu_x_O was ≈140 nm (Figure 1E), which was slightly larger than the size measured by TEM measurement caused by hydration layers. The loading capacity of ABR in NE‐ABR‐Cu_x_O was estimated to be 14.13% by UV–vis spectrophotometry analysis (Figure S2, Supporting Information), and the concentration of Cu in the obtained NE‐ABR‐Cu_x_O solution was 0.03 mg/L determined by inductively coupled plasma‐atomic emission spectrometer (ICP‐AES) analysis (Figure S3, Supporting Information). These quantified values were used for the dose calculation in the following experiments.

### 2.2 Multiple Radical‐Scavenging Capability of NE‐ABR‐Cu_x_O Nanocomplexes

To evaluate the antioxidant ability of NE‐ABR‐Cu_x_O nanocomplexes, multiple representative radicals were included in scavenging assessments, i.e., 2,2‐diphenyl‐1‐picrylhydrazyl radical (DPPH·), 2‐phenyl‐4,4,5,5‐tetramethylimidazoline‐1‐oxyl 3‐oxide radical (PTIO·), hydroxyl radical (·OH), superoxide anion (·O‐ 2), 2,2′‐azino‐bis (3‐ethylbenzothiazoline‐6‐sulfonic acid) radical cation (·ABTS^+^) and hydrogen peroxide (H_2_O_2_). NE‐ABR‐Cu_x_O nanocomplexes displayed high efficiency in scavenging of all examined radicals in a concentration‐dependent manner. For DPPH· (Figure 1F; Figure S4, Supporting Information), PTIO· (Figure 1G), ·OH (Figure 1H), and ·O‐ 2 (Figure 1I), NE‐ABR‐Cu_x_O exhibited potent ROS scavenging under a low concentration of 30 ng/ml (Cu concentration), with inhibition percentages of ≈50%, 31%, 34% and 45%, respectively. Particularly, remarkable inhibition rates of ≈84% and 94% were found for ·ABTS^+^ (Figure 1J) and H_2_O_2_ (Figure 1K) at the same low concentration of NE‐ABR‐Cu_x_O. By comparison, free Cu_x_O nanoparticles exhibited similar inhibition rates for DPPH· (43%), PTIO· (44%), and ·ABTS^+^ (75%) at a significantly higher concentration of Cu (100 ng/mL in free Cu_x_O versus 30 ng mL^−1^ NE‐ABR‐Cu_x_O) in Figure S5 (Supporting Information). The substantially enhanced radical‐scavenging capability of NE‐ABR‐Cu_x_O possibly contributed to the free reductive SH groups in DSPE‐PEG‐SH and improved interaction sites between the coordinated Cu_x_O nanoparticles and free radicals. Collectively, NE‐ABR‐Cu_x_O nanocomplexes are potent anti‐antioxidants with superior performance in scavenging a wide spectrum of radical species.

### 2.3 Protective Effect of NE‐ABR‐Cu_x_O on Primary Cardiomyocytes Against Mitochondrial and DNA Damage

To assess the influence of NE‐ABR‐Cu_x_O on oxidative stress injury, the primary cardiomyocytes were isolated and cultured as previously described,^[^ ^19^ ^]^ the purity of which was verified via the staining of α‐actinin (Figure S6, Supporting Information). For comparison, we constructed nanoemulsions in the absence of therapeutics or in the presence of only ABR or Cu_x_O, which were denoted as NE, NE‐ABR, and NE‐Cu_x_O, respectively. These formulations were similar in sizes (30–200 nm) (Figures S7A–C and S8A–C, Supporting Information). Of note, the TEM image of NE‐Cu_x_O clearly demonstrated that Cu_x_O nanoparticles were uniformly decorated on the surface of nanoparticles (Figure S8B, Supporting Information), implying successful coordination of Cu_x_O and SH groups. The quantified content of Cu in the stock solution was 0.03 µg mL^−1^ measured by ICP analysis (Figure S9, Supporting Information). All nanocomplexes displayed a similar negative surface charge with ζ potential values between −20 and −30 mV (Figure S10, Supporting Information). Fourier transform infrared (FTIR) spectroscopy analysis (Figure S11, Supporting Information) presented the characteristic peak at 770 cm^−1^ (C = C bending band) in the groups of NE‐ABR and NE‐ABR‐Cu_x_O, which was assigned to C = C groups in ABR molecules, verifying the successful encapsulation of ABR. The typical stretching peaks of 1100, 1705, and 2920 cm^−1^ were ascribed to C‐O, C = O, and C‐H bonds from DSPE‐PEG‐SH/ABR in all nanocomplexes.

**Figure 2:**
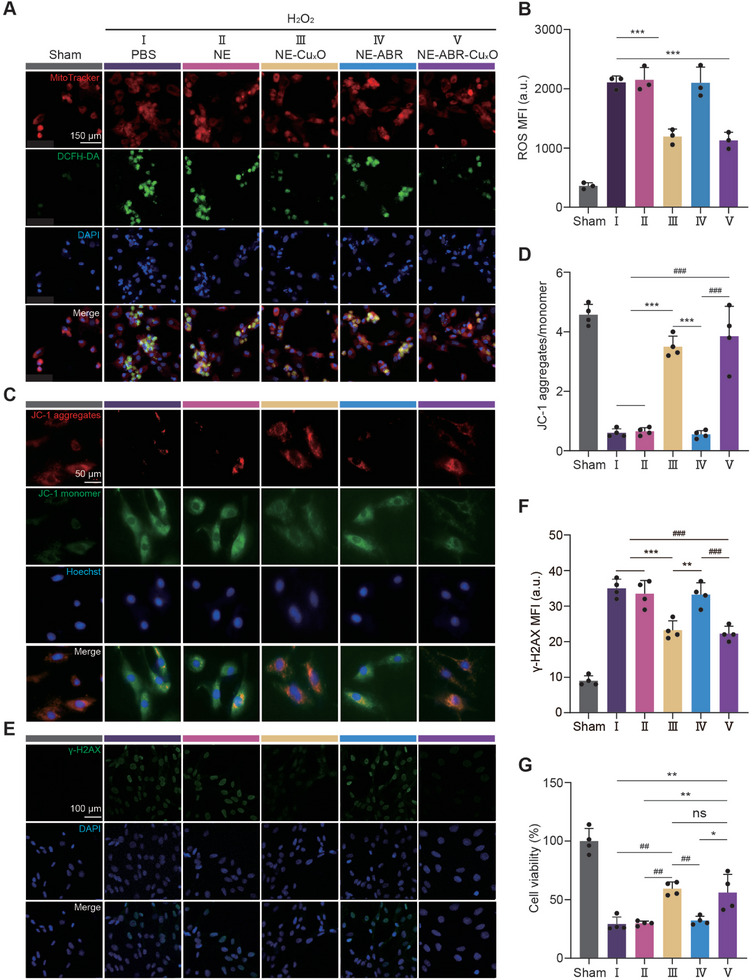
NE‐ABR‐Cu_x_O maintains mitochondrial and DNA functions of cardiomyocytes under oxidative stress. Representative confocal microscopy images A,C,E) and quantitative analysis B,D,F) of intracellular ROS A,B), mitochondrial membrane potential (JC‐1, C‐D), and γ‐H2AX E,F) of primary cardiomyocytes treated with H_2_O_2_ and NE, NE‐ABR, NE‐Cu_x_O, NE‐ABR‐Cu_x_O or PBS. G) Viability of cardiomyocytes under distinct treatments. The statistical difference between the groups was analyzed with the Kruskal‐Wallis test followed by a Dunn's multiple comparisons. Data are represented as mean ± SD. * and # indicted P < 0.05, ** and ## indicted P < 0.01, *** and ### indicted P < 0.001.

H_2_O_2_ was added into the medium (500 µM) for 2 h to mimic the AMI‐induced oxidative stress injury. H_2_O_2_ significantly promoted the production of intracellular ROS, as indicated by ROS probe dichlorodihydrofluorescein diacetate (DCFH‐DA) that was converted into highly green fluorescence DCF upon oxidation (**Figure** **2**A,B). The excessive ROS led to mitophagy, as indicated by MitoTracker (Figure 2A). The treatment of NE‐Cu_x_O or NE‐ABR‐Cu_x_O but not NE or NE‐ABR remarkably scavenged intracellular ROS and alleviated mitophagy. H_2_O_2_ also significantly decreased the mitochondrial membrane potential, as indicated by an increased JC‐1 monomer/JC‐1 aggregate ratio (green/red) (Figure 2C; Figure S12, Supporting Information), which indicated mitochondrial damage. Treatment with NE‐Cu_x_O or NE‐ABR‐Cu_x_O significantly increased the mitochondrial membrane potential compared to the PBS, NE, and NE‐ABR‐treated groups (Figure 2D, all P < 0.001). The restoration of mitochondrial function in cardiomyocytes was attributed to the potent effect of eliminating intracellular ROS. By contrast, the treatment of NE or NE‐ABR showed a limited effect on mitochondrial damage (both P > 0.05), which was caused by the negligible role in ROS scavenging. In addition, the exposure to H_2_O_2_ induced the expression of γ‐H2AX in cardiomyocytes (green, Figure 2E), suggesting DNA damage under oxidative stress. Consistently, NE‐Cu_x_O and NE‐ABR‐Cu_x_O but not NE or NE‐ABR significantly alleviated the H_2_O_2_‐induced DNA damage (Figure 2F). The cell viability assay revealed that H_2_O_2_ significantly decreased the cell viability to ≈25% (Figure 2G), which was dramatically rescued via the treatment of NE‐Cu_x_O or NE‐ABR‐Cu_x_O (Figure 2G, both P < 0.01, compared to the PBS‐treated group), rather than the NE and NE‐ABR (both P > 0.05). In summary, these results confirmed the superior protective effects of NE‐ABR‐Cu_x_O and NE‐Cu_x_O on primary cardiomyocytes against ROS injury.

### 2.4 In Vivo Biosafety and Biodistribution Assessment of NE‐ABR‐Cu_x_O

To evaluate the biosafety of NE‐ABR‐Cu_x_O nanocomplexes, mice were intraperitoneally injected with NE‐ABR‐Cu_x_O every two days over a period of either 7 or 14 days. Blood tests and histological examination of major organs were performed. The results demonstrated that there were no significant differences in white blood cell (WBC), red blood cell (RBC), platelet (PLT), and neutrophil (Neu) between treatment and control groups (Figure S13A–D, Supporting Information). The serum biochemical indices, including creatinine (CREA), blood urea nitrogen (BUN), aspartate aminotransferase (AST), and alanine transaminase (ALT), in mice treated with NE‐ABR‐Cu_x_O nanocomplexes showed similar levels to those in control mice (Figure S13E–H, Supporting Information). The histology analysis of the main organs, including the lung, spleen, liver, heart, and kidney, revealed no apparent lesion evidence of toxicity (Figure S13I, Supporting Information).

To monitor the dynamic biodistribution of NE‐ABR‐Cu_x_O nanocomplexes in vivo, NE‐ABR‐Cu_x_O was labeled with Cy5.5‐ maleimide via maleimide‐thiol reactions, yielding NE‐ABR‐Cu_x_O‐Cy5.5. The mice were sacrificed, and the main organs were harvested at 6 h, 48 h, or 7 days after intraperitoneal injection for in vivo imaging systems (IVIS) analysis. NE‐ABR‐Cu_x_O‐Cy5.5 nanocomplexes were found obviously distributed in the heart and other vital organs, including the liver, spleen, lung, kidney, and gastrointestinaltract at 6 h after injection (Figure S14A, Supporting Information). The distributions in all the vital organs significantly declined at 48 h and Day 7 (Figure S14B, Supporting Information). However, an opposite trend was observed in the kidney, implying the renal retention and clearance of NE‐ABR‐Cu_x_O‐Cy5.5 that might be degraded into nanoparticles less than 10 nm.

Together, these results suggest that NE‐ABR‐Cu_x_O nanocomplexes can effectively reach the therapeutic site in the heart, are systematically and renally clearable, and are well tolerated without obvious systemic toxicity. NE‐ABR‐Cu_x_O nanocomplexes are expected to be a safe therapy for AMI.

### 2.5 Myocardial Function Restoration of NE‐ABR‐Cu_x_O in AMI Mouse Model

**Figure 3:**
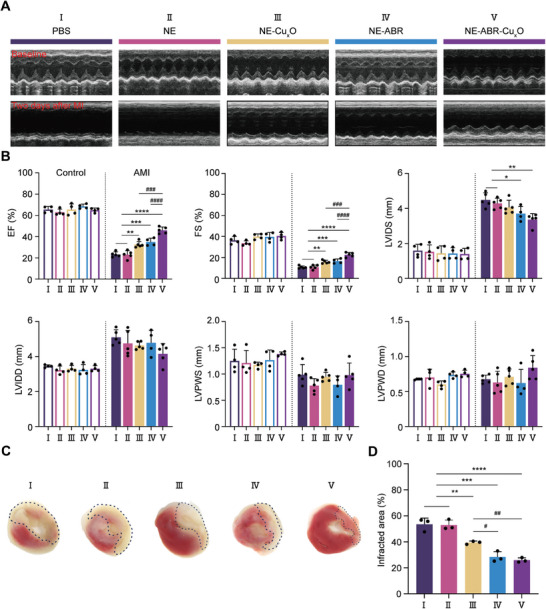
The treatment of NE‐ABR‐Cu_x_O promotes cardiac function and reduces infarction size in AMI mice. A) Representative M‐mode echocardiograms at baseline and 2 days after AMI. B) Cardiac function was assessed by the measurement of EF (%), FS (%), LVIDS (mm), LVIDD (mm), LVPWS (mm), and LVPWD (mm) in AMI mice receiving different treatments. C,D) Representative TTC staining images C) and bar charts D) showed the infarcted sizes in heart harvested 2 days after AMI. The infarction size was defined by the percentage of left ventricular volume (infarcted area %). The statistical difference between the groups was analyzed with the Kruskal‐Wallis test followed by a Dunn's multiple comparisons. Data are represented as mean ± SD. * and # indicted P < 0.05, ** and ## indicted P < 0.01, *** and ### indicted P < 0.001.

To assess the in vivo therapeutic effect of NE‐ABR‐Cu_x_O, AMI was induced by the suture and ligation of the left anterior descending artery (LAD) at a site of 3 mm from its origin post‐surgery in mice. The sham mice underwent the same surgery, except that LAD was not tied. Mice with AMI were treated with NE, NE‐Cu_x_O, NE‐ABR, NE‐ABR‐Cu_x_O, or PBS immediately and one day after the AMI operation. The transthoracic echocardiography was performed before and two days after the AMI operation, and then the mice were sacrificed for triphenyl tetrazolium chloride (TCC) staining. As shown in **Figure** **3**A,B, the transthoracic echocardiography indicated that all nanoformulations mentioned above had no effect on the cardiac function of healthy mice before AMI operation, implying excellent biosafety on myocardial cells. At day 2 post‐AMI, untreated mice showed an apparent decrease in left ventricular ejection fraction (LVEF) and fraction shortening (FS), indicating myocardial dysfunctions. However, the treatment of NE‐Cu_x_O, NE‐ABR, or NE‐ABR‐Cu_x_O but not NE exhibited a significant increase in LVEF and FS compared to untreated AMI mice (Figure 3B), indicating the restoration of myocardial functions. Particularly, the NE‐ABR‐Cu_x_O group showed significant therapeutic improvement compared with the NE‐Cu_x_O and NE‐ABR groups (Figure 3B).

To evaluate the heart structure, left ventricular internal diameter systole (LVIDS), left ventricular posterior wall thickness in diastole (LVPWD), and left ventricle posterior wall thickness in systole (LVPWS) were measured by TTC analysis. Compared to the control, AMI ‐induced LVIDS increased (Figure 3B). The treatment of NE‐Cu_x_O, NE‐ABR, or NE‐ABR‐Cu_x_O but not NE displayed a significant decrease in LVIDS, LVPWD, and LVIDS compared to untreated AMI mice. NE‐ABR‐Cu_x_O showed superior performance in reducing LVIDS than the other two groups, which might be attributed to the combined effect of ABR and antioxidant Cu_x_O. Consistently, a significantly reduced infraction size was detected in NE‐ABR‐Cu_x_O‐treated mice compared to the NE‐Cu_x_O and NE‐ABR groups (Figure 3C,D). Overall, these results demonstrate that NE‐ABR‐Cu_x_O nanocomplexes exhibit powerful cardioprotective effects by enhancing heart function and reducing infarction size in the context of AMI.

### 2.6 Suppression of AMI‐Induced Granulopoiesis Mediated by the Treatment of NE‐ABR‐Cu_x_O

**Figure 4:**
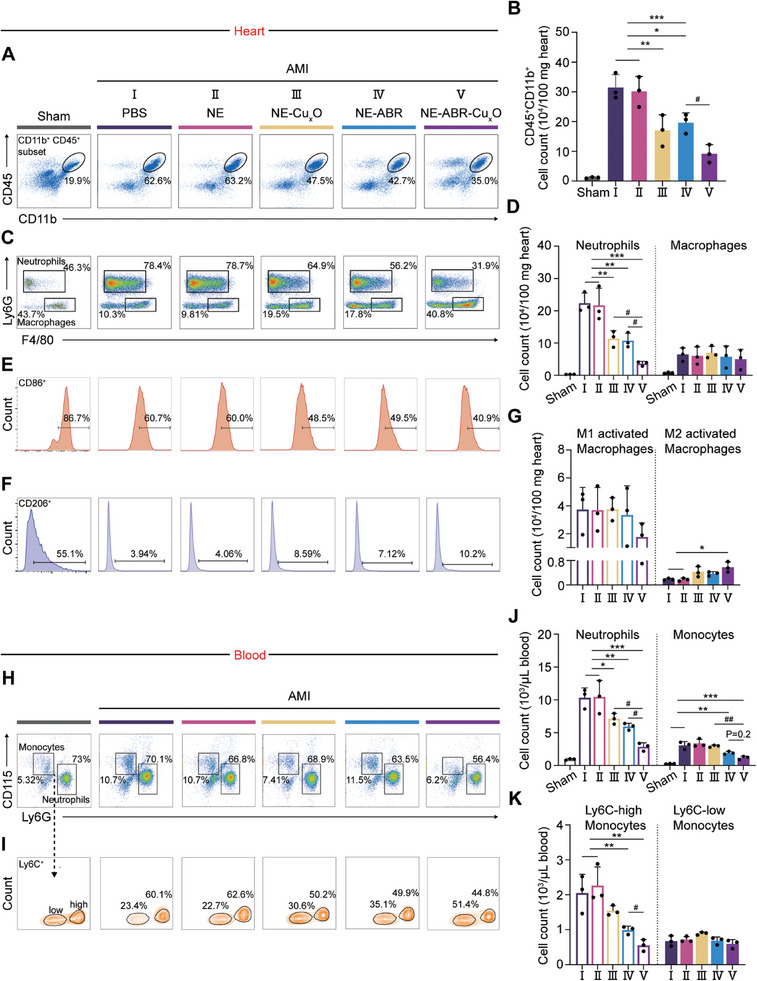
The treatment of NE‐ABR‐Cu_x_O suppresses AMI‐induced granulopoiesis. Flow cytometric analysis shows the representative dot plots (left panel) and quantification (right panel) of neutrophils, monocytes, and macrophages in the heart A–G) and blood H–K) at 2 days post AMI in Sham, AMI and nanocomplexes‐treated AMI mice. Cells in the hearts were normalized to 100 mg of heart tissue. The statistical difference between the AMI groups was analyzed with the Kruskal‐Wallis test followed by a Dunn's multiple comparisons. Data are represented as mean ± SD. * and # indicted P < 0.05, ** and ## indicted P < 0.01, *** and ### indicted P < 0.001.

AMI‐induced granulopoiesis plays an essential role in mediating systemic and local inflammatory responses post ‐AMI, and the neutrophils and macrophages are the main pro‐informatory cells that promote inflammation.^[^ ^20^ ^]^ Previous investigations have proven that ABR regulates AMI‐induced inflammation mainly by inhibiting granulopoiesis. Thus, we performed flow cytometry analysis to investigate the effect of nanocomplexes on granulopoiesis (Figures S15 and S16, Supporting Information). Our data indicated that the treatment of NE‐ABR significantly reduced the frequencies of neutrophils and monocytes infiltrated the heart (**Figure** **4**A–G), indicating the dampening effect on AMI‐induced granulopoiesis. AMI mice were treated with nanocomplexes immediately and one day after the AMI operation, and mice were sacrificed on post‐operative day two. Meanwhile, the treatment of NE‐Cu_x_O displayed a comparable therapeutic effect in inhibiting the infiltration of neutrophils and monocytes. Strikingly, NE‐ABR‐Cu_x_O showed the most potent effect on reducing infiltrated neutrophils in the heart (Figure 4C,D). Regarding the macrophages in the heart, all the above treatments did not reduce the infiltrating CD11b^+^ F4/80^+^ Ly6G^−^ macrophages, while the subtype analysis (Figure 4E–G) suggested that NE‐ABR‐Cu_x_O significantly promoted the population of CD86^+^ M2‐type macrophages (anti‐inflammatory) but not CD206^+^ M1‐type macrophages (pro‐inflammatory). By contrast, NE‐ABR and NE‐Cu_x_O did not alter the frequencies of both phenotypic macrophages.

Consistently, CD115^+^ Ly6G^−^ monocytes and CD115^−^ LyG6^+^ neutrophils circulating in the blood showed a similar trend in number reduction after each intervention (Figure 4H,J; Figure S17, Supporting Information). As shown in Figure 4J, the monocytes in the blood were significantly reduced in the NE‐Cu_x_O group (P < 0.01) and NE‐ABR‐Cu_x_O group (P < 0.001), as compared to the PBS‐ and NE‐treated groups. NE‐ABR‐Cu_x_O‐treated mice displayed further decreased monocytes when compared to the NE‐Cu_x_O group (P < 0.01). Further phenotyping analysis indicated that the inhibition of the proinflammatory monocytes (Ly6C‐high monocytes) contributed to the decreased number of total monocytes (Figure 4I–K), while no significant differences regarding the number of anti‐inflammatory monocytes (Ly6C‐low monocytes) were found among all the groups (Figure 4K, all P > 0.05). These results implied that NE‐ABR‐Cu_x_O nanocomplexes played remarkable anti‐inflammatory effects compared to a single treatment by effectively suppressing the infiltration of pro‐inflammatory subsets of innate immune cells.

### 2.7 NE‐ABR‐Cu_x_O does not Target the BONE marrow to Inhibit the AMI‐Induced Granulopoiesis

Despite the fact that previous investigations have proven that ABR targets cardiac neutrophils to inhibit AMI‐induced granulopoiesis,^[^ ^4a^ ^]^ we observed that the immune cell counts in the above experiment diminished in both the heart and the blood. The question raised whether the target was the infarcted heart or the bone marrow (BM). Next, we sought to answer this question by tracking the distribution of NE‐ABR‐Cu_x_O‐Cy5.5 in BM post‐administration. Figure S18 (Supporting Information) showed no noticeable fluorescence signals of NE‐ABR‐Cu_x_O‐Cy5.5 in BM, indicating that the nanocomplexes did not enter BM. This result essentially ruled out the possibility that NE‐ABR‐Cu_x_O inhibited the egress of immune cells from the hematopoietic niches during AMI. In addition, we further conducted experiments to check whether NE‐ABR‐Cu_x_O could inhibit the pharmacologically induced release of immune cells from the BM (Figure S19a, Supporting Information). The results demonstrated that granulocyte colony‐stimulating factor (G‐CSF) significantly promoted the release of neutrophils from the BM in the blood (Figure S19b, Supporting Information) but not BM (Figure S19c,d, Supporting Information). However, the NE‐ABR‐Cu_x_O made no significant effects on the G‐CSF‐induced promotion of neutrophils (P > 0.05). Overall, the therapeutic effect of NE‐ABR‐Cu_x_O in alleviating granulopoiesis w achieved by targeting the infarcted heart rather than the BM.

### 2.8 Potent Inhibition Effect of NE‐ABR‐Cu_x_O on Myocardial Apoptosis and Inflammation in AMI Mice

**Figure 5:**
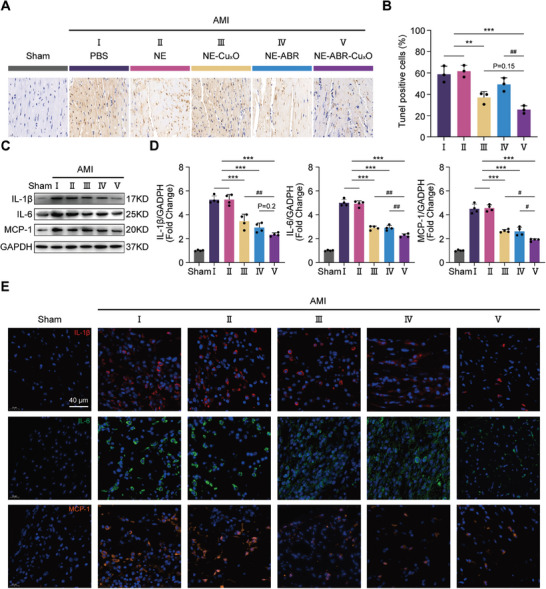
NE‐ABR‐Cu_x_O nanocomplexes inhibit apoptosis and inflammation in AMI heart. A) Representative TUNEL staining images and B) the statistical difference of TUNEL positive cells in heart tissues at 2 days post AMI from Sham, AMI, and nanocomplexes‐treated AMI mice. C,D) Western blotting and E) representative immunofluorescence staining images of pro‐inflammatory cytokines, IL‐1β, IL‐6 and MCP‐1 in AMI hearts from mice receiving distinct treatments as indicated (the quantitative results seen in Figure S21, Supporting Information). The statistical difference was analyzed with the Kruskal‐Wallis test followed by a Dunn's multiple comparisons. Data are represented as mean ± SD. * and # indicted P < 0.05, ** and ## indicted P < 0.01, *** and ### indicted P < 0.001.

After AMI, ROS are released immediately by ischemic cardiomyocytes, promoting the expression of chemokines and pro‐inflammation cytokines, leading to apoptosis and necrosis of cardiomyocytes and, eventually, the decline in cardiac function. The proinflammatory cytokines, interleukin 6 (IL‐6), IL‐1β, and MCP‐1(monocyte chemoattractant protein‐1), play a central role in AMI pathophysiology,^[^ ^21^ ^]^ thus we next explored the changes of these key chemokines and cytokines via western blotting (WB) and immunohistochemistry at 2 days after AMI, to understand the therapeutic mechanism of NE‐ABR‐Cu_x_O. AMI‐induced apoptosis (detected by TUNEL staining) was significantly ameliorated after the treatment of NE‐Cu_x_O (NE‐Cu_x_O group versus PBS and NE groups, both P < 0.01) (**Figure** **5**A,B; Figure S20, Supporting Information). A significant difference was detected between the NE‐ABR‐Cu_x_O and NE‐ABR group, and a similar tendency was found between NE‐ABR‐Cu_x_O and NE‐Cu_x_O, suggesting that the significant improvement of NE‐ABR‐Cu_x_O in protecting myocardial cells from apoptosis was contributed by the combined therapeutic effect from ABR and Cu_x_O.

Regarding the expression of inflammatory cytokines, WB analysis (Figure 5C,D) indicated that the treatments of NE‐ABR‐Cu_x_O, NE‐ABR, and NE‐Cu_x_O significantly decreased the protein expression of IL‐1β, IL‐6, and MCP‐1. Among these treatment groups, NE‐ABR‐Cu_x_O showed a superior inhibition effect on the expression of these inflammatory cytokines as compared to the NE‐ABR and NE‐Cu_x_O groups. The immunofluorescence analysis (Figure 5E; Figure S21, Supporting Information) suggested a similar trend in cytokine expression with WB results, further supporting the potent anti‐inflammation effect of NE‐ABR‐Cu_x_O nanocomplexes.

### 2.9 Long‐Term Ameliorating Effect of NE‐ABR‐Cu_x_O on Progressive Heart Remodeling and Loss of Function Post‐MI

**Figure 6:**
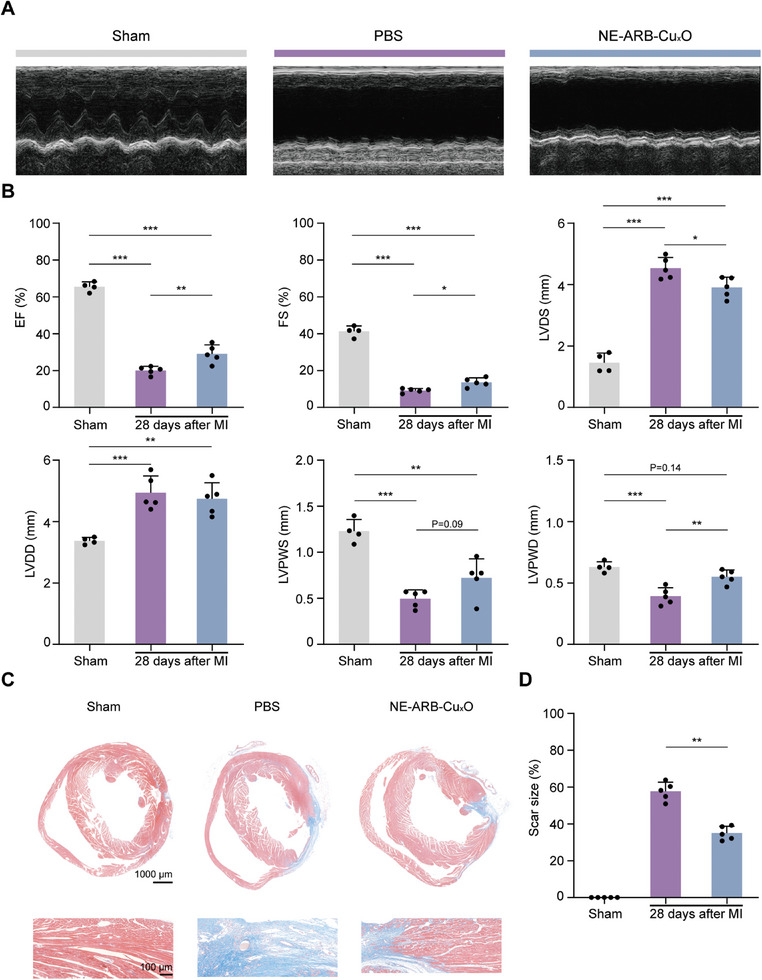
NE‐ABR‐Cu_x_O nanocomplexes exhibit a long‐term inhibition effect on the loss of cardiac function and myocardial fibrosis post MI. A) Representative echocardiograms at 28 days after MI. B) Cardiac function was assessed by the measurements of EF%, FS%, LVIDS (mm), LVIDD (mm), LVPWS (mm) and LVPWD (mm). C) Representative images and D) statistical difference of myocardial fibrosis sizes stained by Masson staining at 28 days after AMI. The statistical difference was analyzed with the Kruskal‐Wallis test followed by a Dunn's multiple comparisons. Data are represented as mean ± SD. * indicted P < 0.05, ** indicted P < 0.01, *** indicted P < 0.001.

To validate the long‐term therapeutic efficacy of NE‐ABR‐Cu_x_O, we next examined the ventricular remodeling (Masson staining for myocardial fibrosis) and changes in cardiac function (transthoracic echocardiography) at 28 days after MI. The nanocomplexes were injected immediately after surgery and once per day within the three post‐operative days. The results demonstrated that the treatment of NE‐ABR‐Cu_x_O nanocomplexes significantly relieved the long‐term decline in EF and FE percentages (**Figure** **6**A,B), implying durable restoration of cardiac functions. Similarly, NE‐ABR‐Cu_x_O nanocomplexes maintained a marked reduction in LVIDS, LVIDD, LVPWS, and LVPWD, suggesting a longstanding effect on heart remodeling. Pathological examination revealed that the chronic myocardial fibrosis size in the heart was significantly reduced after the intervention of NE‐ABR‐Cu_x_O (Figure 6C,D). Collectively, these results verified the long‐term therapeutic effect of NE‐ABR‐Cu_x_O with dramatic promotion of heart remodeling and function restoration, representing a potent therapeutic modality with clinical potential for treating AMI diseases.

### 2.10 Molecular Mechanism Studies of NE‐ABR‐Cu_x_O using the Transcriptomic Analysis

**Figure 7:**
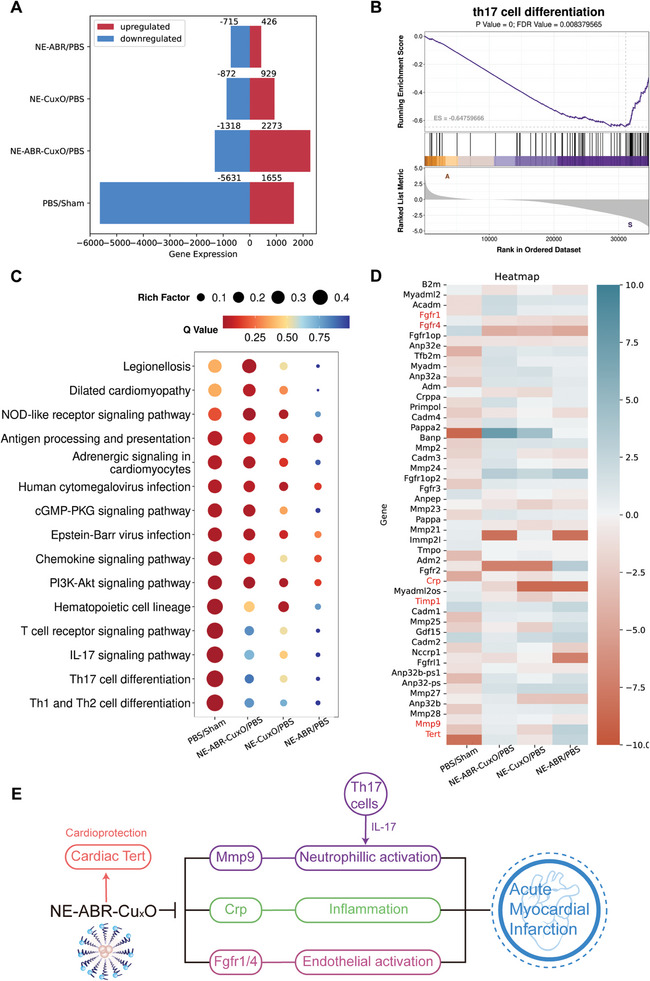
Transcriptome profiles of untreated and treated infarcted hearts reveal potential therapeutic mechanism. A) Up‐ and down‐regulated DEG in different groups. B) GSEA depicts the significant Th17 cell differentiation mediated inflammatory pathway in infarcted hearts treated with PBS. C) KEGG map shows the most down‐regulated inflammatory pathways, particularly IL‐17 signaling pathway and Th17 cell differentiation, in NE‐ABR‐Cu_x_O treated group by comparting with untreated group in infarcted hearts. D,E) A heatmap D) shows expression of MI related genes in infarcted hearts with distinct treatments. Red marks the genes correlated with neutrophilic activation, inflammation, endothelial activation, cardiac Tert activation E).

To reveal the detailed mechanisms underlying the protective action of NE‐ABR‐Cu_x_O on the infarcted hearts, RNA sequencing analysis was performed. Compared to the control group (PBS‐treated group), 7286 differently expressed genes (DEGs) were detected. In the Sham group, 1655 genes were up‐regulated, while the remaining 5631 genes were down‐regulated (**Figure** **7**A). By contrast, the DEG numbers were obviously decreased with only 1801 DEGs (929 up‐regulated and 872 down‐regulated genes), 1141 DEGs (426 up‐regulated and 715 down‐regulated genes), and 3591 DEGs (2273 up‐regulated and 1318 down‐regulated genes) between NE‐Cu_x_O versus PBS, NE‐ABR versus PBS, and NE‐ABR‐Cu_x_O versus PBS, respectively.

For further understanding of the immunological regulation by NE‐ABR‐Cu_x_O on AMI, Gene Scores Enrichment Analysis (GSEA) and Kyoto Encyclopedia of Genes and Genomes (KEGG) pathway enrichment analysis were conducted (Figure 7B,C). The DEGs between the NE‐ABR‐Cu_x_O and PBS group were significantly enriched in the Th17 differentiation pathway (Figure 7B), which represented a trigger of the proliferation and recruitment of neutrophils by secreting IL‐17 cytokines.^[^ ^22^ ^]^ After the treatment with NE‐ABR‐Cu_x_O, NE‐Cu_x_O, or NE‐ABR, Th17 cell differentiation and IL‐17 signaling pathways, as well as other inflammation‐ and neutrophils‐ related pro‐inflammatory pathways were downregulated, including the nod‐like receptor signaling, chemokine signaling and Th1/Th2 cell differentiation signaling pathways.

Previous investigations have identified a significant increase in fibroblast growth factor receptor (FGFR) 1 and FGFR 4,^[^ ^23^ ^]^ and the inflammatory factors such as c‐reactive protein (CRP),^[^ ^24^ ^]^ tissue inhibitors of metalloproteinases (TIMPS)^[^ ^25^ ^]^ and matrix metalloproteinase‐9 (MMP9) after MI,^[^ ^26^ ^]^ all of which exacerbated the inflammation to worsen the cardiac function. According to the heatmap analysis (Figure 7D), the FGFR1/4 and the inflammatory factors were detected as being down‐regulated after the treatment of NE‐ABR, NE‐Cu_x_O, or NE‐ABR‐Cu_x_O. Notably, NE‐ABR‐Cu_x_O treatment exhibited the strongest regulatory effect on these genes among the three nanocomplex groups, representing one of the mechanisms by which NE‐ABR‐Cu_x_O exerted cardioprotection after MI. Beyond that, the telomerase (TERT) that was recently validated as a protective factor after MI,^[^ ^27^ ^]^ was highly expressed in NE‐ABR‐Cu_x_O and NE‐ABR groups but not NE‐Cu_x_O, implying a new therapeutic targeting pathway of ABR on cardioprotection.

Collectively, the RNA sequencing analysis revealed the detailed mechanism by which the NE‐ABR‐Cu_x_O down‐regulates the genes related to neutrophilic activation, endothelial activation, and inflammation while upregulates the genes associated with cardiac Tert activation, thereby protecting cardiac functions after AMI with improved ventricular function and reduced infarct scars (Figure 7E).

## 3 Discussion

Post MI, cardiomyocyte damage triggers the production of massive ROS via the mitochondrial oxidative phosphorylation, which promotes the influx of high frequencies of proinflammatory phenotypic immune cells at the infracted area, mainly including neutrophils, monocytes and macrophages circulated from the bloodstream.^[^ ^6^ ^]^ Excessive inflammation aggravates cardiac ischemic injury and heart failure. Among the cardiac immune cells, macrophages resident in the infracted areas are mainly pro‐inflammatory M1 type, modulating the inflammatory responses. The application of antioxidant nanomedicines is one of the most attractive strategies for alleviating oxidative stress‐mediated heart injury. For example, allomelanin nanoparticles were engineered to effectively scavenge multiple free radicals, which thereby promoted the shift of M1 to M2 type macrophages, and reduced the infiltration of neutrophils.^[^ ^28^ ^]^ As the first responders, neutrophils secret alarmins of S100A8/A9, which primes the TLR4‐inflammasome‐IL‐1β signaling pathway in naïve neutrophils, fueling the infiltration of other subsets of innate immune cells.^[^ ^4a^ ^]^ Thus, recently emergent nanoplatforms targeting neutrophil‐driven pathologies are an alternative appealing approach for treating AMI.^[^ ^29^ ^]^

Despite the advancements in anti‐inflammation nanotherapeutics, single‐target nanomedicines often find it hard to fully revere the progression of AMI, which is caused by the multiplicity of targets orchestrating the elevated pathological inflammation. To overcome the heterogenicity of the inflammatory network, our work modulates a broad spectrum of targets (ROS and neutrophils) using one immuno‐nanoplatform. Referring to previously reported studies in the field of conjoint therapeutic nanoplatforms,^[^ ^30^ ^]^ we set up saline, empty NP, NP‐Cu_x_O, and NP‐ABR as the control groups. Our data indicated that the NE‐ABR‐Cu_x_O displayed the strongest therapeutic effects in AMI compared to all the other groups, supporting the enhanced effects of Cu_x_O and ABR loaded by NE‐ABR‐Cu_x_O. In addition to the expected therapeutic targets, the innovative combination of antioxidant Cu_x_O nanoparticles and S100A8/A9 inhibitor (ABR) molecules suggests new mechanisms for restoring the functions of cardiomyocytes. Our results imply that the immunocomplexes downregulate the Th17‐IL‐17 signaling pathway, thereby reducing neutrophil activities. Direct targeting the upstream of Th17 cells might offer advanced treatment modalities. Beyond conferring effects on reversing pathological inflammation and immunity, the nanocomplexes containing ABR display a potential function in direct TERT activation ‐mediated cardioprotection. The discovered heterogenous and complex network of endothelia‐cardiomyocyte‐immune cells‐cytokines (Figure 7E) provides new insights into AMI treatment.

In summary, we developed multifaceted immunomodulatory NE‐ABR‐Cu_x_O nanocomplexes for the treatment of AMI. NE‐ABR‐Cu_x_O was found to effectively alleviate oxidative stress by scavenging multiple free radicals of DPPH·, PTIO·, ·OH, ·ABTS^+^, ×O‐ 2, and H_2_O_2_. In addition, the developed nanocomplexes enabled specific regulation of neutrophilic inflammation by blocking S100A8/A9‐NLPR3‐IL‐1β signals, thereby potently suppressing the infiltration of massive proinflammatory innate immune cells post AMI. Particularly, NE‐ABR‐Cu_x_O therapy contributed to a dramatic decrease in the frequencies of Ly6C‐high monocytes and neutrophils while a significant increase in the proportion of anti‐inflammatory phenotypic M2 macrophages. The combination of immunomodulatory ABR and antioxidant Cu_x_O nanoparticles substantially improved the therapeutic effect in restoring the function of damaged myocardial cells and reducing fraction size in AMI compared to mono‐ABR or Cu_x_O ‐mediated therapy. RNA sequencing analysis uncovered the detailed potential gene network and new therapeutic cardiac Tert activation pathway. Our work offers a promising strategy to integrate enzyme ‐mimicking nanomedicine therapy with immunotherapy for durable, effective, and specific treatment of AMI, holding substantial potential for clinical translation. In follow‐up studies, we attempt to optimize the method and timing of NE‐ABR‐Cu_x_O administration, which may represent one way to further improve the therapeutic efficacy.

## 4 Experimental Section

### Chemical Reagents

S100A8/A9 inhibitor (ABR215757, paquinimod) was purchased from the MCE company (MedChemExpress). DSPE‐PEG‐SH was purchased from the BroadPharm company. Copper (II) chloride dihydrate (CuCl_2_×2H_2_O) was bought from General Reagent of Titan (Shanghai, China). L‐ascorbic acid (AA), sodium hydroxide (NaOH), and N,N‐dimethylformamide (DMF) were ordered from Sinopharm Chemical Reagent (Shanghai, China).

### Preparation and Characterization of NE‐ABR‐Cu_x_O

*Cu_x_O nanoparticles*. The Cu_x_O was synthesized according to the previous report.^[^ ^13b^ ^]^ First, 10 mmol CuCl_2_×2H_2_O powder was dissolved in 50 mL deionized water. Subsequently, a slow dropwise addition of 0.1 M L‐AA solution (50 mL) was initiated. The pH of the solution was then adjusted to 8–9 by 1 M NaOH. The resulting mixture was continuously stirred at 80 °C in an oil bath for a duration of 12 h. Afterward, the supernatant was collected by centrifugation at 8000 rpm for 15 min. The obtained solution was subjected to dialysis (Mw cutoff: 3000) for one day, purifying Cu_x_O nanoparticles.

### NE‐ABR‐Cu_x_O

To prepare therapeutics‐loaded nanoemulsions, 30 mg DSPE‐PEG‐SH and 6 mg ABR were dissolved in 1 mL DMF. The solution was subjected to ultrasonication until it became colorless and transparent. Subsequently, the DMF solution was dropwise added to 4 mL deionized water or phosphate‐buffered saline (PBS) containing 100 ng of Cu_x_O nanoparticles. The mixture was stirred for a duration of 12 h. Following the incubation period, the reaction mixture was dialyzed (Mw cutoff: 3000) for 24 h.

### NE‐ABR‐Cu_x_O‐Cy5.5

To achieve fluorescence labeling of NE‐ABR‐Cu_x_O, Cy5.5‐maleimide (Life‐iLab, AP35L084) and DSPE‐PEG‐SH were dissolved in DMF with a molar ratio of 1:10 to form DSPE‐PEG‐SH‐Cy5.5 via maleimide‐thiol reactions. NE‐ABR‐Cu_x_O‐Cy5.5 was prepared as described in the fabrication of NE‐ABR‐Cu_x_O by replacing DSPE‐PEG‐SH with DSPE‐PEG‐SH‐Cy5.5.

### Free Radical Scavenging Assessment of NE‐ABR‐Cu_x_O

The free radical scavenging ability of NE‐ABR‐Cu_x_O was detected with various antioxidant activity assays and monitored by UV–vis spectroscopy, including H_2_O_2_ assay, ·O‐ 2 assay, ·OH assay, DPPH· assay, PTIO· assay, and ·ABTS^+^ assay. The inhibition percentage of radicals was calculated as follows except for ·OH:

(1) $$\mathrm{Inhibition}\;\;\left(\%\right)=\frac{A0-A}{A0}\;\times 100\%\;$$

where A is the absorbance of NE‐ABR‐Cu_x_O after incubation with various indicators, and A0 is the absorbance of a blank control that replaced NE‐ABR‐Cu_x_O with deionized water.

### DPPH

1 mg of DPPH was dissolved in 24 mL of anhydrous ethanol and ultrasonicated for 5 min to ensure complete dissolution. Subsequently, DPPH solution (1 mL) was mixed with anhydrous ethanol (0.5 mL), and the absorbance measured at 519 nm by a microplate reader was recorded as A0. Then, DPPH solution (1 mL) was mixed with varying concentrations of NE‐ABR‐Cu_x_O (6‐30 ng mL^−1^), and after incubation at 37 °C for 30 min, the absorbance was measured and recorded as A.

### PTIO

3 mg of PTIO was dissolved in deionized water (30 mL) under ultrasonication for 5 min to ensure complete dissolution. Subsequently, the prepared PTIO solution (0.8 mL) was mixed with deionized water (0.2 mL) for dilution, and then the absorbance measured at 557 nm was recorded as A0. Then, DPPH solution (0.8 mL) was mixed with varying concentrations of NE‐ABR‐Cu_x_O and then incubated at 37 °C for 2 h. Afterward, the absorbance was measured and recorded as A.

### OH

27.8 mg of FeSO_4_·7H_2_O and 18 mg of 1,10‐Phenanthroline (PHEN) were separately dissolved in deionized water (50 mL). Subsequently, 1 mL of the FeSO_4_·7H_2_O solution, 0.2 mL of various concentrations of NE‐ABR‐Cu_x_O, and 1 mL of 400 µM H_2_O_2_ were incubated in the dark for 3 min. Afterward, 1 mL of phenanthroline was added, and the reaction proceeded in the dark for 30 min. Finally, the absorbance was measured at 510 nm and recorded as A. In the same procedure, when NE‐ABR‐Cu_x_O was replaced with water, the obtained value was recorded as Ac, and when H_2_O_2_ was replaced with water, the obtained value was recorded as A0. Therefore, the formula for calculating the inhibition percentage was as follows:

(2) $$\mathrm{Inhibition}\;\;\left(\%\right)=\frac{A-\mathrm{Ac}}{A0-\mathrm{Ac}}\;\times 100\%$$

### ABTS^+^

3 mg of ABTS diammonium salt was dissolved in 0.735 mL of deionized water, and 1 mg of K_2_S_2_O_8_ was separately dissolved in 1.43 mL of water. Subsequently, 0.2 mL of each of the above solutions was mixed and allowed to stand in the dark for 12 h. Afterward, it was diluted 20–30 times with anhydrous ethanol, and the diluted solution (0.8 mL) was mixed with anhydrous ethanol (0.2 mL). The absorbance was then measured at 734 nm and recorded as A0. After mixing with different concentrations of NE‐ABR‐Cu_x_O, the absorbance was measured and recorded as A.

### O‐ 2

125 mM of L‐methionine, 750 µM of nitrotetrazolium blue chloride, and 20 µM of riboflavin were prepared. Subsequently, 0.3 mL of each solution was mixed with 1.8 mL of PBS (pH 7.4). Afterward, the resulting mixture was combined with varying concentrations of NE‐ABR‐Cu_x_O and placed in a light incubator for 15 min. The absorbance was then measured at 550 nm and recorded as A.

### H_2_O_2_

According to a previous report,^[^ ^31^ ^]^ 2 g KI and 1 g potassium hydrogen phthalate were separately dissolved in 30 and 48 mL of deionized water, respectively. 20 mM H_2_O_2_ solution was then prepared. Subsequently, 0.9 mL of water, 35 µL of H_2_O_2_, and 0.1 mL of NE‐ABR‐Cu_x_O were mixed and incubated at 37 °C for 2 h. Afterward, 0.1 mL of KI and potassium hydrogen phthalate were added, and the mixture was left to develop color for 30 min. The absorbance was then measured at 350 nm and recorded as A.

### In Vitro Experiments

*Cell models*: The primary cardiomyocytes were obtained and cultured as previously described.^[^ ^19^ ^]^ One‐day‐old SD rat was quickly sacrificed, and the hearts were digested with 0.03% trypsin and 0.04% collagenase type II. Subsequently, the cardiomyocytes were isolated and seeded in culture plates, the purity of which was verified via the immunofluorescence staining of α‐actinin. To mimic the AMI‐induced oxidative injury, 500 µM H_2_O_2_ was added into the medium for 2 h.

### Detection of Mitochondrial Function

The mitochondrial morphology was detected via MitoTracker Red CMXRos staining according to the manufacturer's instructions (Invitrogen, M7512). The function of the mitochondrial respiratory chain and the antioxidant efficiency of nanocomplexes were measured by JC‐1 staining according to the manufacturer's instructions (Thermo Fisher, M34152). Briefly, the cardiomyocytes were incubated in DMEM containing PBS, NE‐ABR, NE‐Cu_x_O (50 ng Cu_x_O/mL) or NE‐ABR‐Cu_x_O (50 ng Cu_x_O/mL) for 1 h and then H_2_O_2_ was added. After further incubating for 2 h, the medium was replaced with DMEM containing MitoTracker Red CMXRos and Hoechst 38 450, or JC‐1 and Hoechst 38 450. Following 30 min incubation, the cells were further photographed with a confocal microscope (ZEISS, LSM710) or analyzed by flow cytometry.

### Assessment of Intracellular ROS

In another parallel experiment, the DCFH‐DA was used to evaluate the intracellular ROS according to the instructions (Sigma–Aldrich, D6883). Briefly, the intracellular ROS oxidizes non‐fluorescent DCFH to fluorescent form. Therefore, the fluorescent intensity indicates the level of intracellular ROS. After the aforementioned incubation, the cells were washed, and then 10 µM DCFH‐DA was added to the medium for further incubation in the dark for 30 min. Afterward, the cells were washed and imaged or subjected to a flow cytometry (Cytek Biosciences, Fremont, CA) to quantify the intracellular ROS levels respectively.

### Measurement of DNA Injury

In another parallel experiment, the injury of DNA was measured by γ‐H2AX staining according to the instructions (Beyotime, C2035S). After the treatment described above, the cardiomyocytes were fixed and blocked and then incubated with anti‐γ‐H2AX primary antibody overnight. Following washing, the cells were incubated with the secondary antibody at room temperature for one hour.

### Evaluation of Cell Viability

In another parallel experiment, the viability of cardiomyocytes was measured by MTT Assay (Beyotime, C0009S). After the above‐mentioned treatments, the cells were incubated with an MTT solution for 4 h. After the dark blue formazan crystals were dissolved, the absorbance was determined at 570 nm. Finally, the cell viability was shown as the treated group/control group ratio (%).

### Mice and Treatments

The adult male C57BL/6J mice aged 8–10 weeks were purchased from the Shanghai SLAC Laboratory. The experiments were approved by the Animal Care and Use Committee of Shanghai Chest Hospital (KS(Y)22 241) and were performed in accordance with the National Institutes of Health Guide for the Care and Use of Laboratory Animals. Mice were intraperitoneally injected (i.p.) with PBS, NE‐ABR (8 mg ABR/kg), NE‐Cu_x_O (4 µg Cu_x_O/kg), or NE‐ABR‐Cu_x_O (8 mg ABR/kg and 4 µg Cu_x_O/kg) nanocomplexes immediately after surgery and once per day within postoperative three days. PBS served as the vehicle control.

### Assessment of In Vivo Distribution

C57BL/6J mice were intraperitoneally injected with NE‐ABR‐Cu_x_O‐Cy5.5 (8 mg ABR/kg and 4 µg Cu_x_O/kg) and then sacrificed at 6 h, 48 h and 7 days. The organs and lower limb bones were dissected from mice treated with ABR‐Cu_x_O‐Cy5.5 or PBS for *ex vivo* imaging using IVIS. Finally, the fluorescence images and intensity were acquired by Living Image (Perkin Elmer).

### Biosafety Evaluation

The biosafety evaluation was conducted in C57BL/6J mice administrated with PBS or NE‐ABR‐Cu_x_O nanocomplexes (8 mg ABR/kg and 4 µg Cu_x_O/kg). The mouse blood and major organs were collected at 7 days or 14 days after administration. The blood routine test of red blood cell (RBC), white blood cell (WBC), platelet (PLT), and neutrophil (Neu) was performed, and the major organs were collected for histology studies. Meanwhile, the blood was centrifuged at 1500 rpm for 10 min for further analysis of the levels of CK, AST, ALT, BUN, and CREA in serum.

### Mouse Model of Myocardial Infarction

At surgery, the mice were anesthetized with sodium pentobarbital (60 mg kg^−1^, i.p.) and maintained using 1% isoflurane. Briefly, after the heart was exposed, the myocardial ischemia was induced by the suture and ligation of LAD at a site of 3 mm from its origin. The sham mice underwent the same procedure, with the exception that the LAD was not tied.

### Transthoracic Echocardiography

The transthoracic echocardiography (Biosound Esaote Inc.) was performed at the indicated time points to evaluate cardiac function, and five mice were subjected to the measurements in each group. The left ventricle parameters were measured at both the long‐ and short‐axis views. End‐systole or end‐diastole was achieved when the LV size was the smallest or largest, respectively. LVESD and LVEDD were measured using M‐mode tracing. The percentage of FS was calculated as (LVEDD‐LVESD)/LVEDD × 100%.

### Measurement of Myocardial Infarction Sizes and Scar Sizes

TTC staining in the acute phase. The heart tissue was harvested two days after MI. After the section, the slices were immediately immersed in 2% TTC at 37 °C for 30 min. The infarcted heart appeared TTC‐negative (pale) after the staining and was outlined for further calculation via ImageJ software. The infarcted area% was calculated as the ratio of TTC‐negative area/total LV wall area × 100%. Masson's trichrome staining in chronic phase. The heart was harvested at four weeks after MI. After the routine process, the heart sections (5 mm thick) were stained with Masson's trichrome to measure the cardiac fibrosis and collagen deposition as previously described. The images were acquired digitally and measured using ImageJ.

### Flow Cytometry

*Heart tissue*: The single ‐cell suspension was prepared as previously described to analyze the leukocytes in the heart. Briefly, the heart was harvested, cleared of adhering tissues and atria, minced into three pieces, transferred to GentleMACS C tubes containing collagenase type II (1 mg mL^−1^), and processed into a fine suspension using a tissue dissociator (GentleMACS Octo Dissociator, Miltenyi Biotec). The suspension was filtered through a 100 µm strainer, rinsed with FACS buffer, and centrifuged at 500 g for 7 min at 4 °C. The resultant pellet was resuspended in 1 mL buffer to determine cell count, followed by staining for Live/Dead marker and surface proteins as described above.

Data were acquired on an NL‐CLC flow cytometer (Cytek Biosciences, Fremont, CA), and the analysis was performed with FlowJo software (Ashland, OR). After excluding the doublets (by FSC‐H versus FSC‐A) and dead cells (Live versus Dead), the neutrophils were identified as CD45^+^, CD11b^+^, Ly6G‐hi cells, and the macrophages as CD45^+^, CD11b^+^, Ly6G^−^. To identify the polarization state, the macrophages were further classified as CD86^+^ cells (M1 activated macrophages) and CD206^+^ cells (M2 activated macrophages). The gating strategy was shown in Figure S12 (Supporting Information).

### Peripheral Blood Leukocytes

The red blood cell lysed cells were washed and resuspended in 100 µL of FACS buffer containing a cocktail of various antibodies directed against different types of leukocytes. The stained cells were tested on an NL‐CLC flow cytometer, and the data was analyzed via FlowJo software. The neutrophils were identified as CD45^+^, CD11b^+^, and Ly6G^+^ cells, and the monocytes were identified as CD45^+^, CD11b^+^, Ly6G^−^, and CD115^+^ cells, which were further classified as pro‐inflammatory Ly6C‐hi monocytes and anti‐inflammatory Ly6C‐low monocytes. The gating strategy was shown in Figure S13 (Supporting Information).

### Immunofluorescence Assay

Immunofluorescence staining was performed to detect the expression of IL‐1β, IL‐6, and MCP‐1 in formalin‐fixed sections of the infarcted heart. The preparation of the sections was the same as in our previous study.^[^ ^19^ ^]^ The prepared sections were incubated with the primary antibody at 4 °C overnight (IL‐1β:Abcam, ab254360, 1:100; IL‐6:Abcam, ab290735, 1:100; MCP‐1:Thermo Fisher, MA5‐17040, 1:300). After incubating with secondary antibody (IL‐1β:Abmart, M21014, 1:1000; IL‐6:Abmart, M21012, 1:1000; MCP‐1:Abmart, M213408, 1:1000) for one hour at RT, the sections were washed and stained with DAPI. The images were obtained by confocal photography.

### Western Blotting

The procedure for protein extraction and the Western blotting experiment was the same as the previous experiment. The antibodies used were listed as following: IL‐1β‐Abmart, PK56327, 1:500; IL‐6‐Abmart, TD6087, 1:1000; MCP‐1‐Abmart, TD7577, 1:1000; secondary antibody‐Abmart, M21002, 1:5000.

### RNA Sequencing

The infarcted hearts were harvested two days after an infarction, and Trizol extracted the total RNA. In the present study, the DESeq algorithm to conduct the differential expression analysis was applied, and the FDR‐corrected P < 0.05, log2FC > 1, or log2FC < −1 was identified for the threshold for differentially expressed genes. To further explore the functional and biological implications of the DEGs, the GSEA and KEGG enrichment analyses were conducted.

### Evaluation of the Effect on the Release of Neutrophils from the Bone Marrow

To perform the pharmacologically induced release of neutrophils from the bone marrow, the mice were subcutaneously injected with a daily dose of 250 µg kg we applied of recombinant human G‐CSF protein (Proteintech, HZ‐1207) for 7 days.^[^ ^32^ ^]^ NE‐ABR‐Cu_x_O was i.p. injected simultaneously once per day to check whether it inhibits the G‐CSF‐induced release. Four hours after the last injection, the mice were sacrificed to obtain peripheral blood and bone marrow for further analysis. The count of neutrophils in blood was assessed by the routine blood tests. The bone marrow was prepared as described previously and analyzed by flow cytometry.^[^ ^4a^ ^]^ After excluding the doublets (by FSC‐H versus FSC‐A) and dead cells (Indo 1^+^), the neutrophils were identified as CD45^+^ CD115^−^, Gr‐1^+^ cells.

### Statistical Analysis

All data were presented as mean ± SD. For comparing the differences between the two groups, the Mann‐Whitney test (U test) was applied. For the comparisons of three or more groups, the Kruskal‐Wallis test, followed by Dunn's multiple comparisons, was used. The P value ≤ 0.05 was considered statistically significant. All analyses were performed via SPSS version 22.0, and the detailed analysis method was described in each figure legend.

## Conflict of Interest

The authors declare no conflict of interest.

## Supporting information

Supporting Information

## Data Availability

The data that support the findings of this study are available from the corresponding author upon reasonable request.
