# Supplementary material for: Immuno‐Nanocomplexes Target Heterogenous Network of Inflammation and Immunity in Myocardial Infarction
Source: Adv Sci (Weinh). 2024 Jul 25;11(36):2402267. doi: 10.1002/advs.202402267 (PMC11423151; doi:10.1002/advs.202402267)
Supplement: Supplementary file 1 — Supporting Information [file ADVS-11-2402267-s001.docx]

Supporting Information

**Immuno-nanocomplexes Target Heterogenous Network of Inflammation and Immunity in Myocardial Infarction**

*Fan Su^#^, Weifan Ye^#^, Yi Shen^#^, Yujie Xie, Chong Zhang, Qianyun Zhang, Zhengqi Tang, Meihua Yu*, Yu Chen*, Bin He**

*^#^These authors contributed equally to this work*

**Supplementary figures**


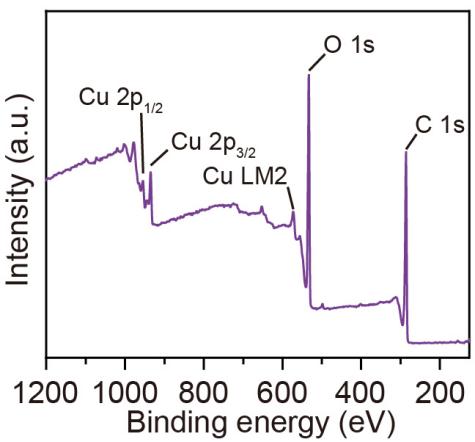


**Figure S1.** The XPS survey spectrum of Cu_x_O nanoparticles.


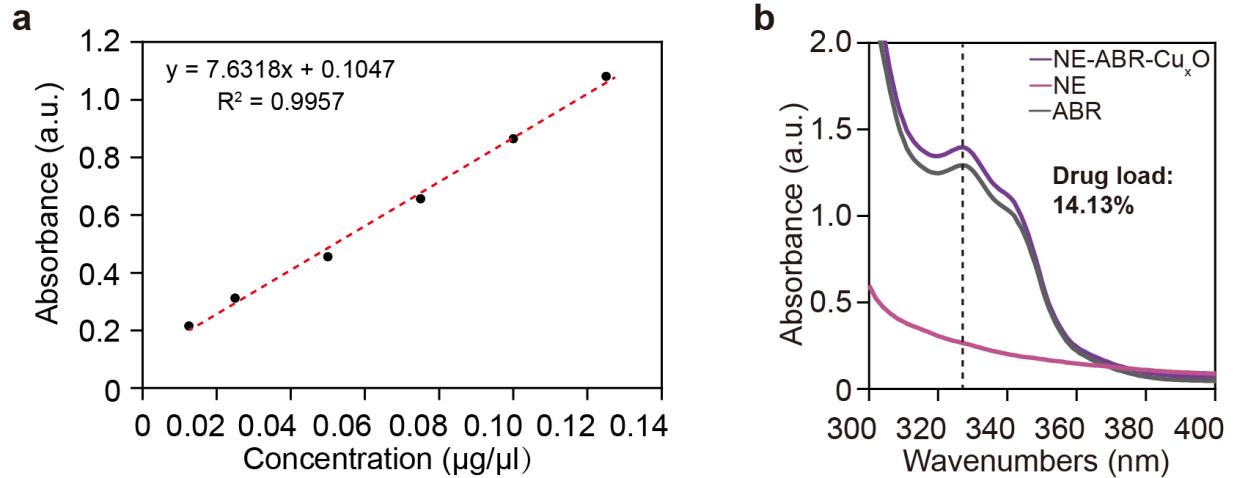


**Figure S2.** (a) The standard curve of paquinimod (ABR) measured at 327 nm by UV-vis. (b) UV-vis spectra of freeze-dried NE, NE-ABR-Cu_x_O and free ABR dissolved in DMF solution.


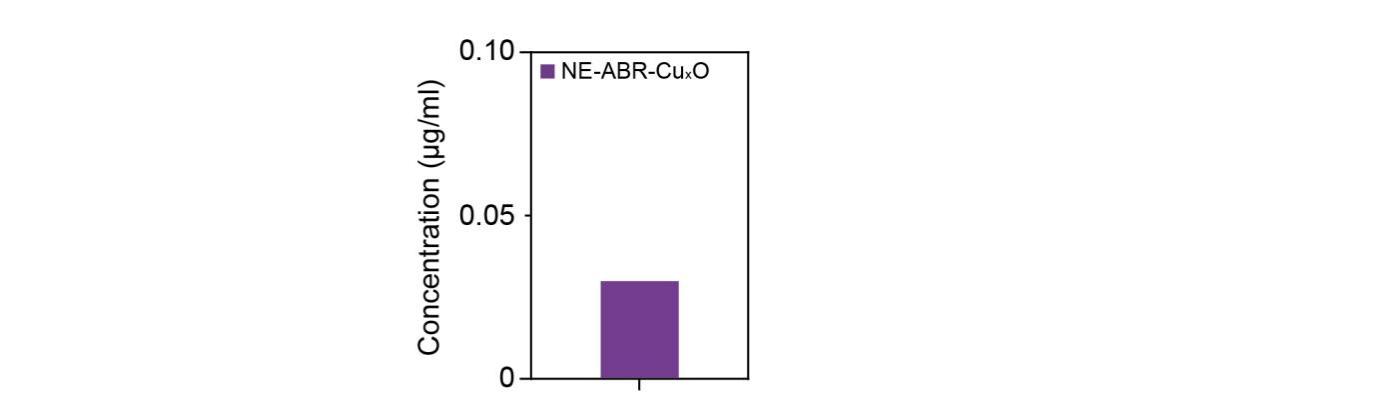


**Figure S3.** Quantitative analysis of Cu of NE-ABR-Cu_x_O by ICP-AES.


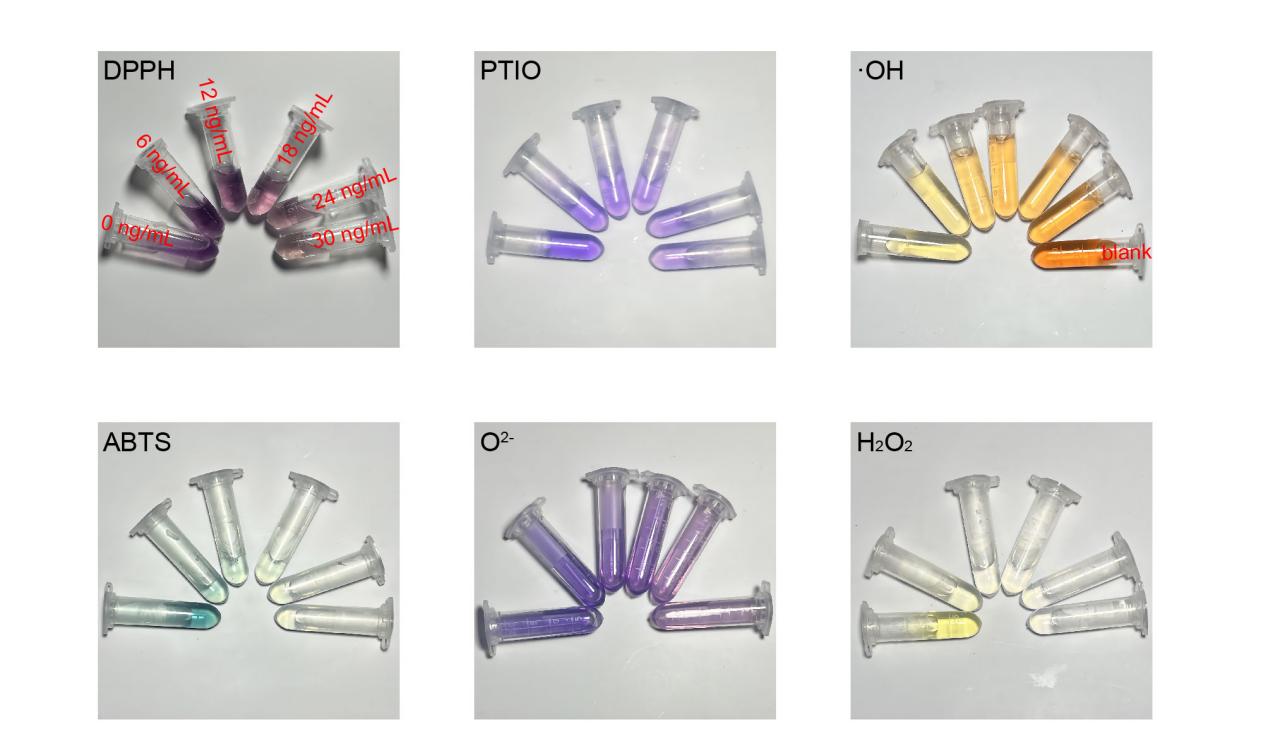


**Figure S4.** Digitial photos show the the color changes of different free-radicals under coincubation with various concentration of NE-ABR-Cu_x_O.


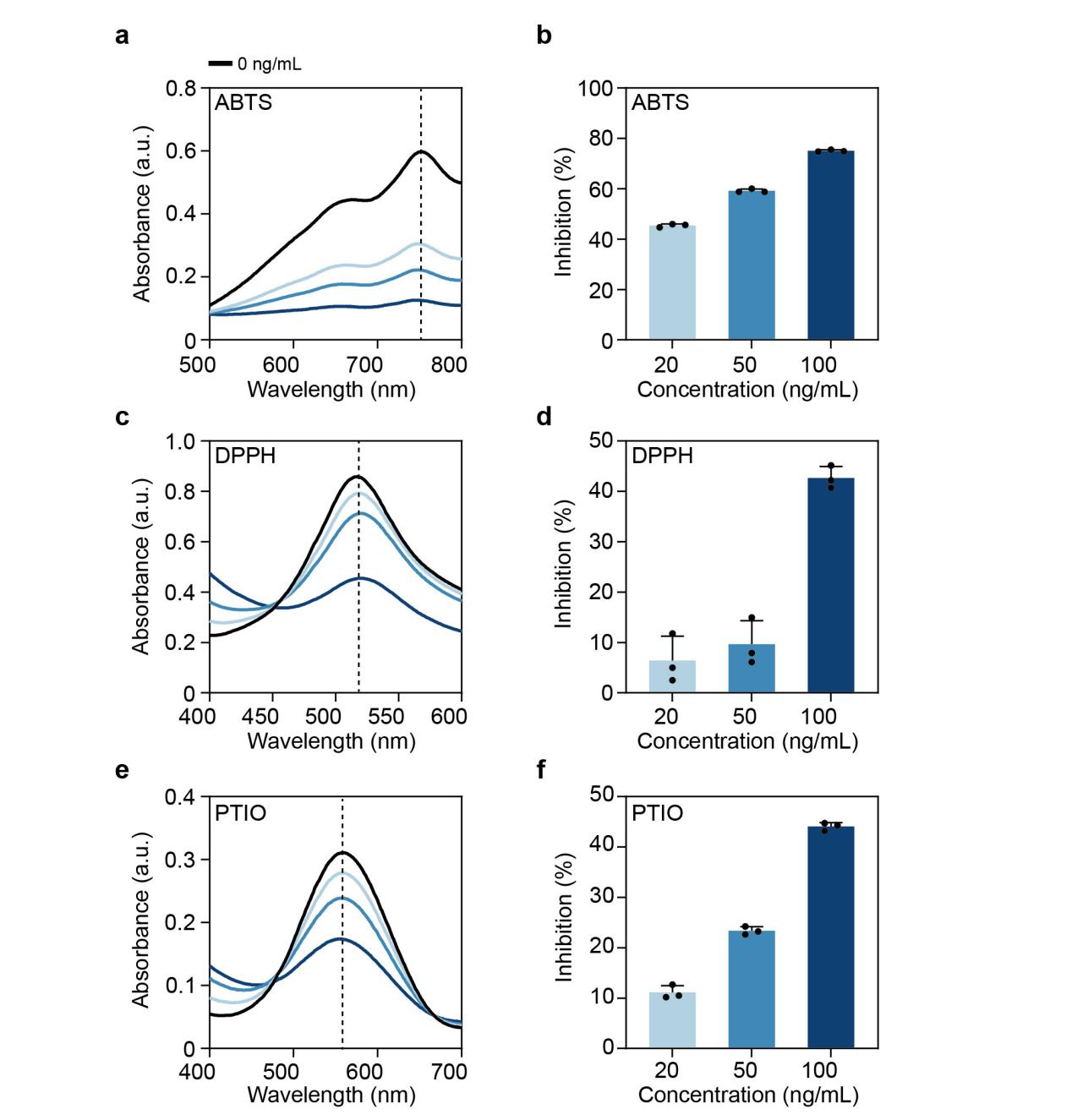


**Figure S5.** The free-radical scavenging ability of Cu_x_O nanoparticles was monitored via UV−vis spectroscopy and quantitatively analyzed: (a,b) ABTS, (c,d) DPPH, (e,f) PTIO.


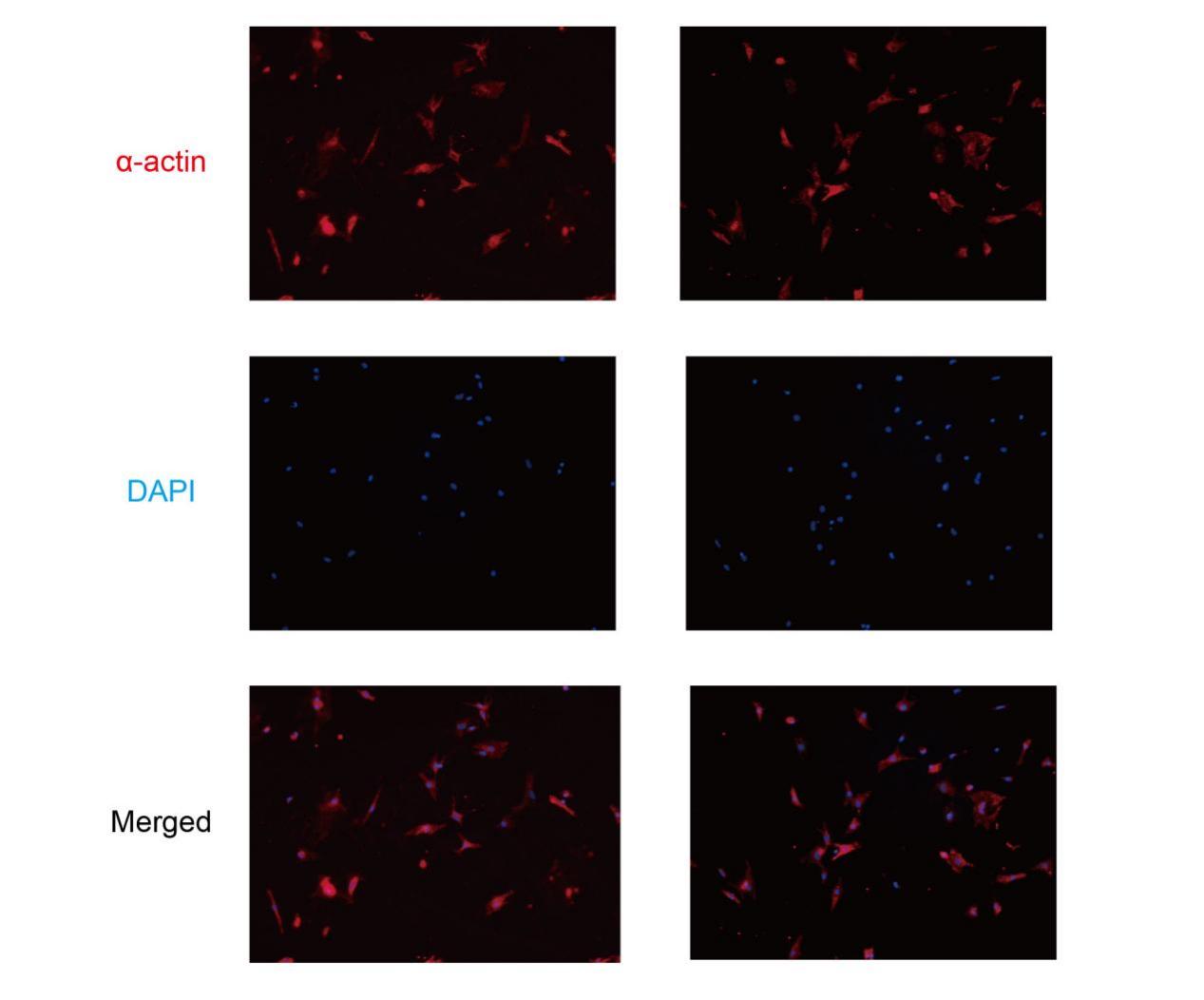


**Figure S6.** The purity of primary cardiomyocytes was confirmed by immunofluorescence staining of α-actinin (red).


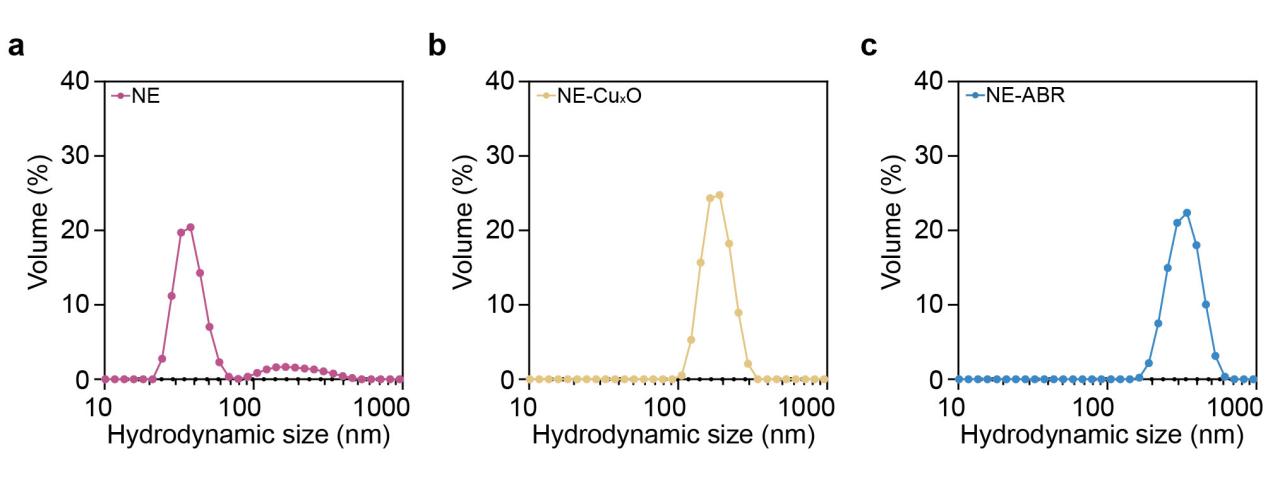


**Figure S7.** Hydrodynamic diameter distribution curves of (a) NE, (b) NE-Cu_x_O, and (c) NE-ABR.


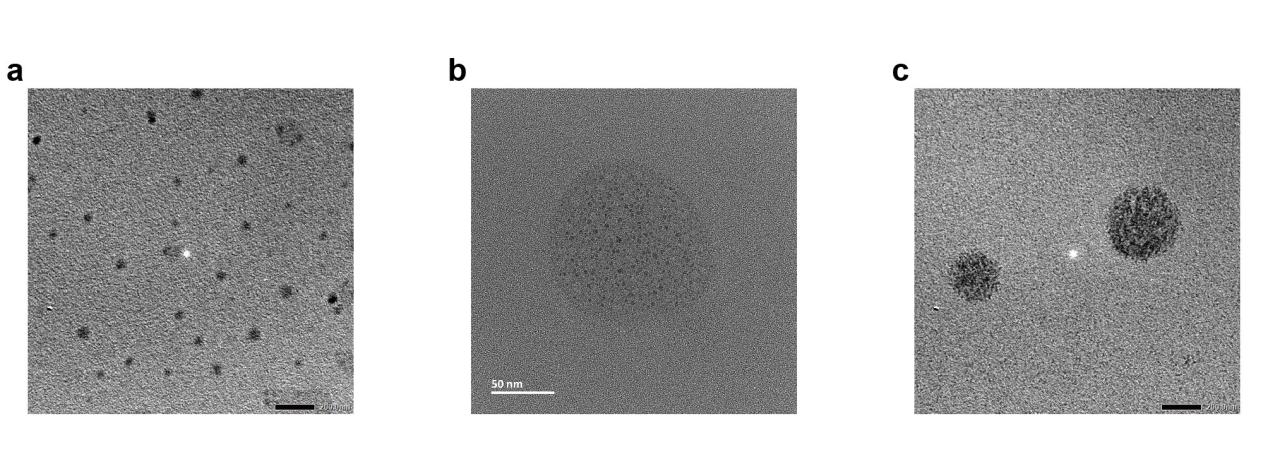


**Figure S8.** TEM images of (a) NE (a, scale bar: 200 nm), (b) NE-Cu_x_O (b, scale bar: 50 nm), (c) NE-ABR (c, scale bar: 200 nm). The samples of NE and NE-ABR were positively stained with uranyl acetate before TEM observation.


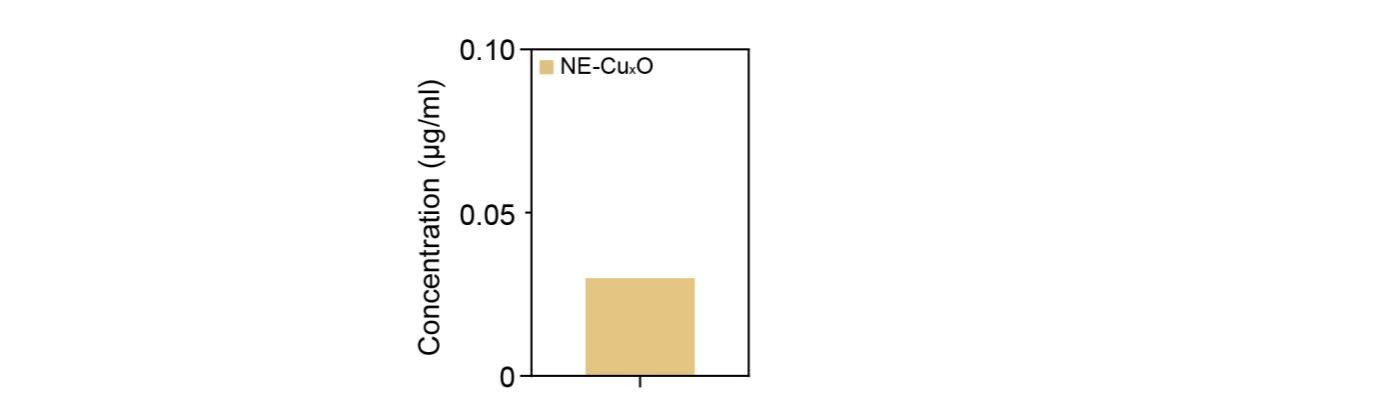


**Figure S9.** Quantitative analysis of Cu of NE-Cu_x_O by ICP-AES.


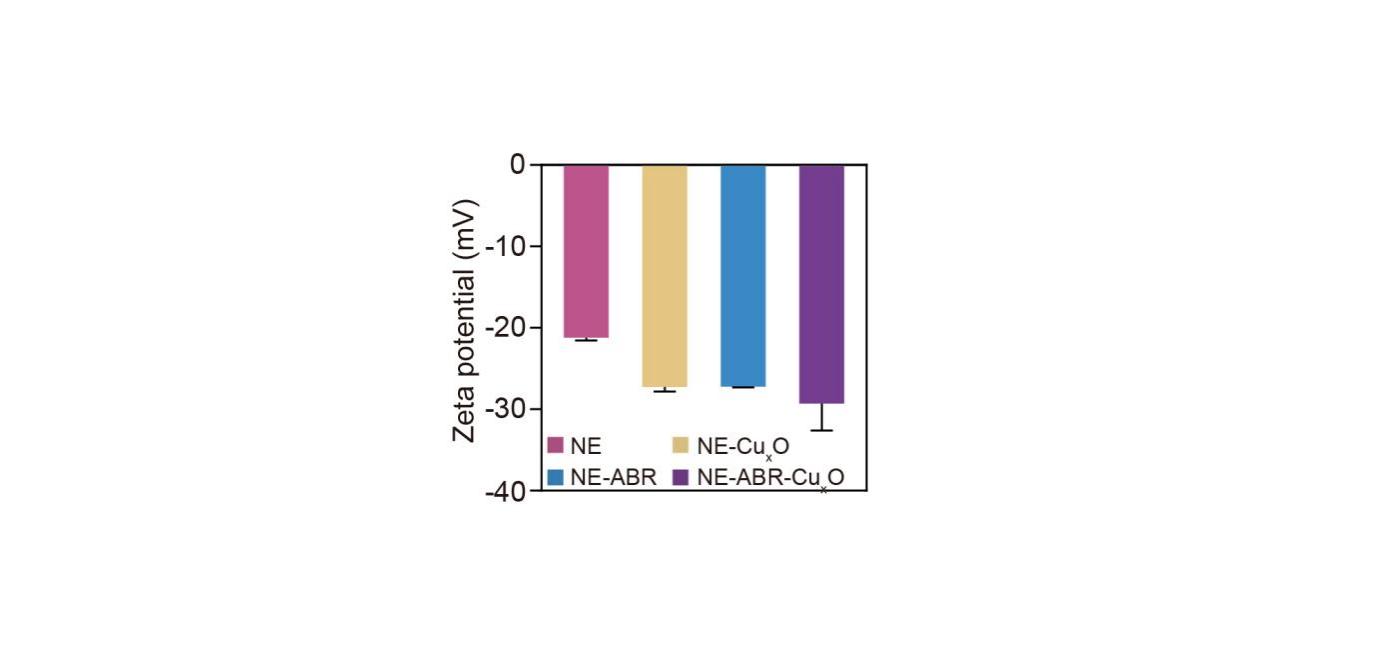


**Figure S10.** Zeta potential values of NE, NE-Cu_x_O, NE-ABR and NE-ABR-Cu_x_O.


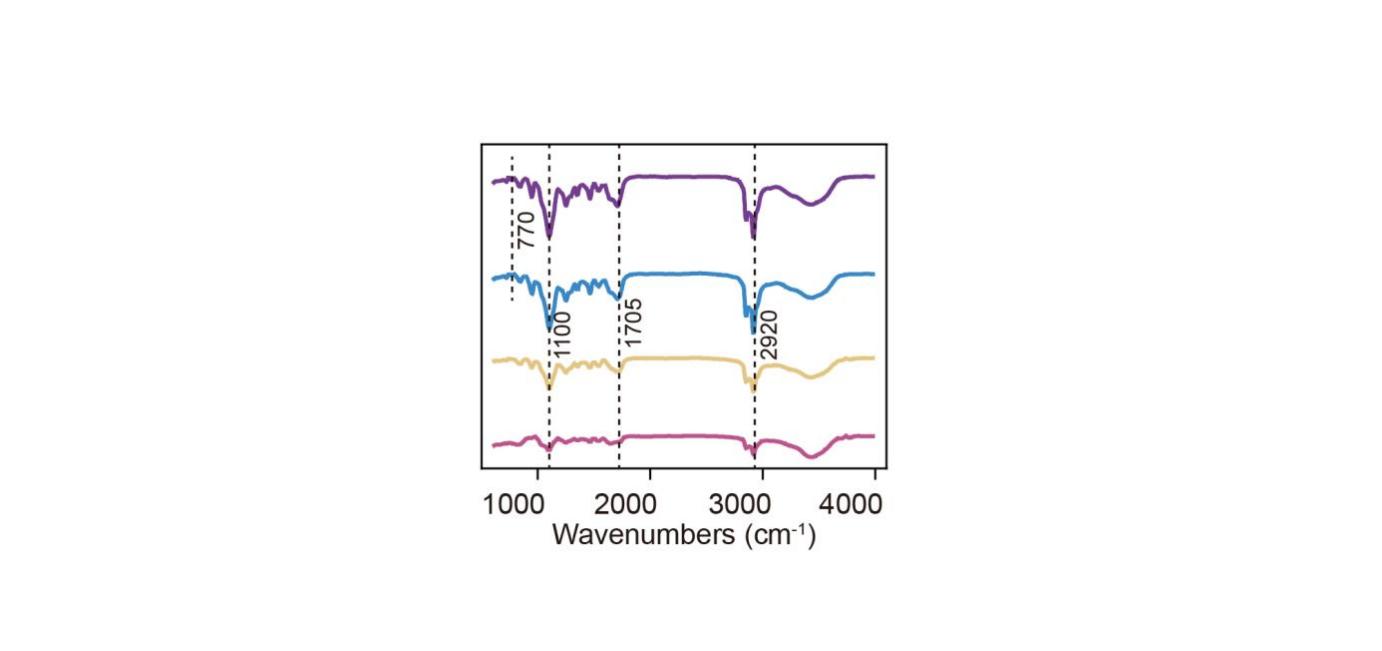


**Figure S11.** FTIR spectra of NE, NE-Cu_x_O, NE-ABR and NE-ABR-Cu_x_O.


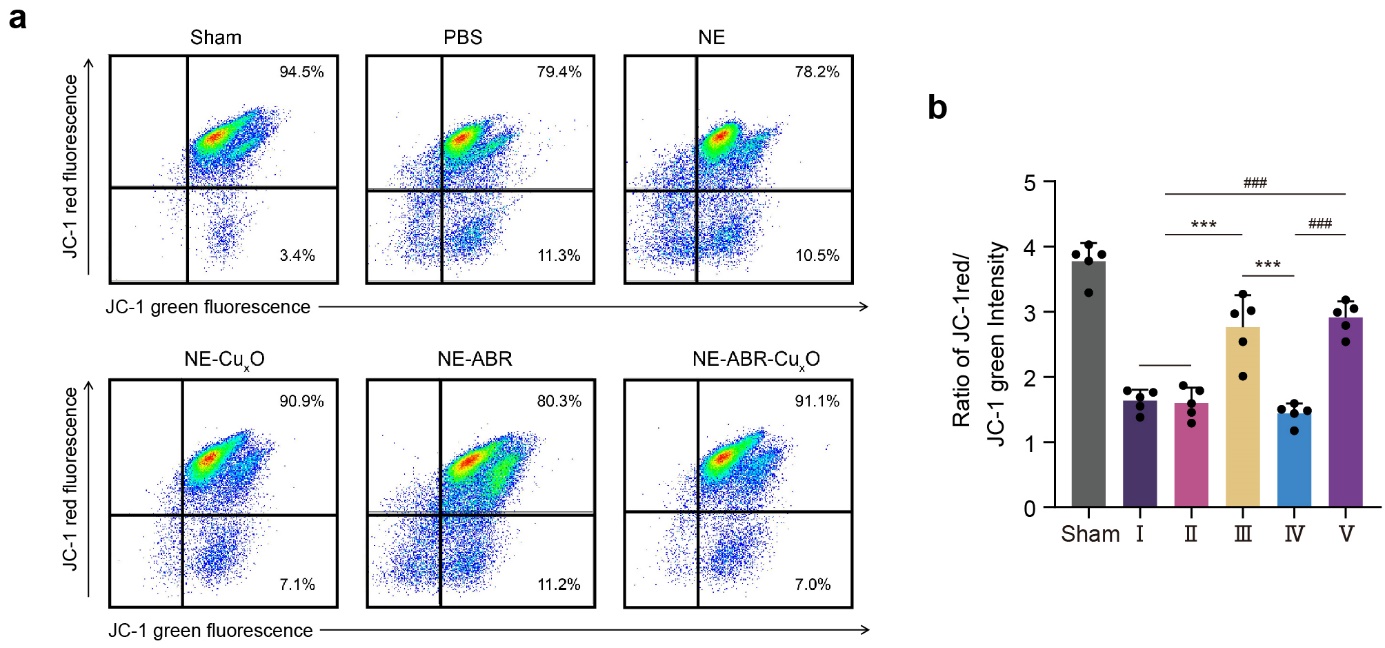


**Figure S12.** (a) Representative flow cytometric dot plots and (b) bar charts showing H_2_O_2_ pre-treated primary cardiomyocytes with JC-1 staining after different treatments as indicated.


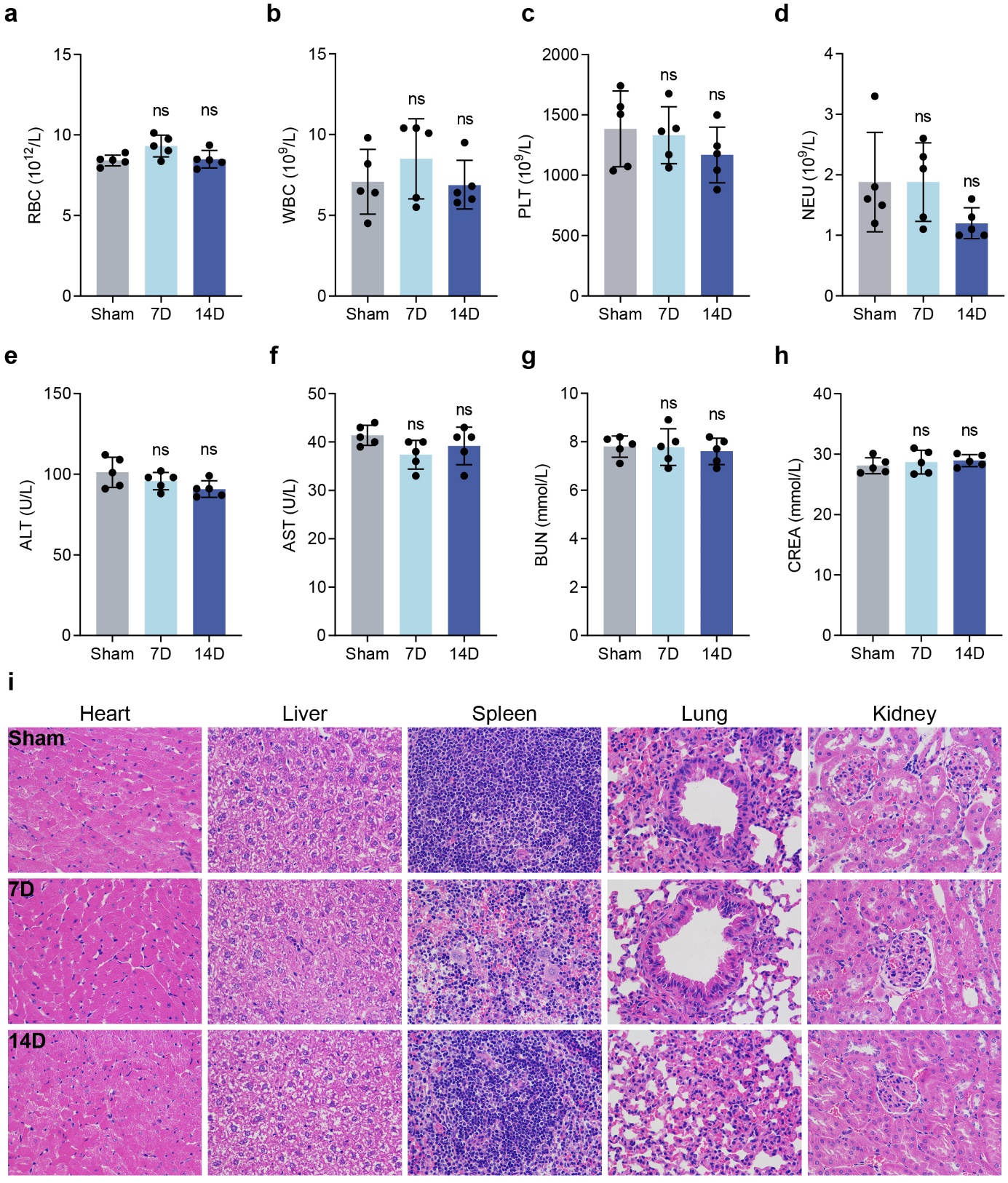


**Figure S13.** Biosafety analyses of NE-ABR-Cu_x_O. (a-d) Routine blood tests, (e-h) serum biochemical analysis and (i) representative H&E staining images of heart, liver, spleen, lung and kidney from mice at 7 days (7D) and 14 days (14D) post-injection of NE-ABR-Cu_x_O (n = 5).


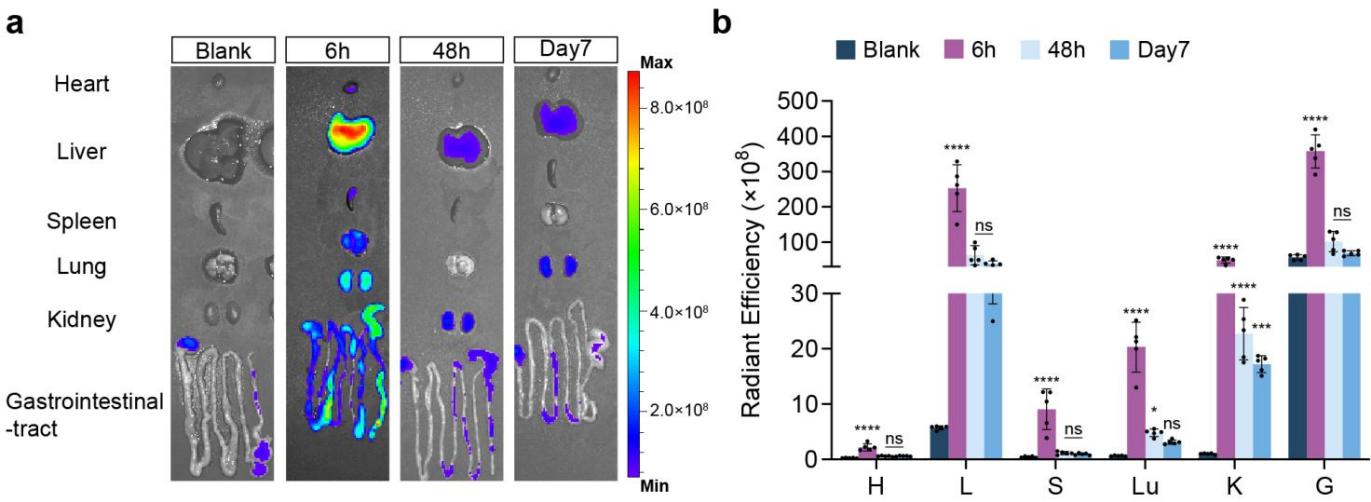
**Figure S14.** Representative *ex vivo* fluorescent images (a) and quantitative analysis (b) (n = 5) of organs derived from C57BL/6 male mice at 6 h, 48 h and 7 days after intraperitoneal injection with NE-ABR-Cu_x_O-Cy5.5. H: heart, L: liver, S: spleen, Lu: lung, K: kidney and G: gastrointestinal-tract.


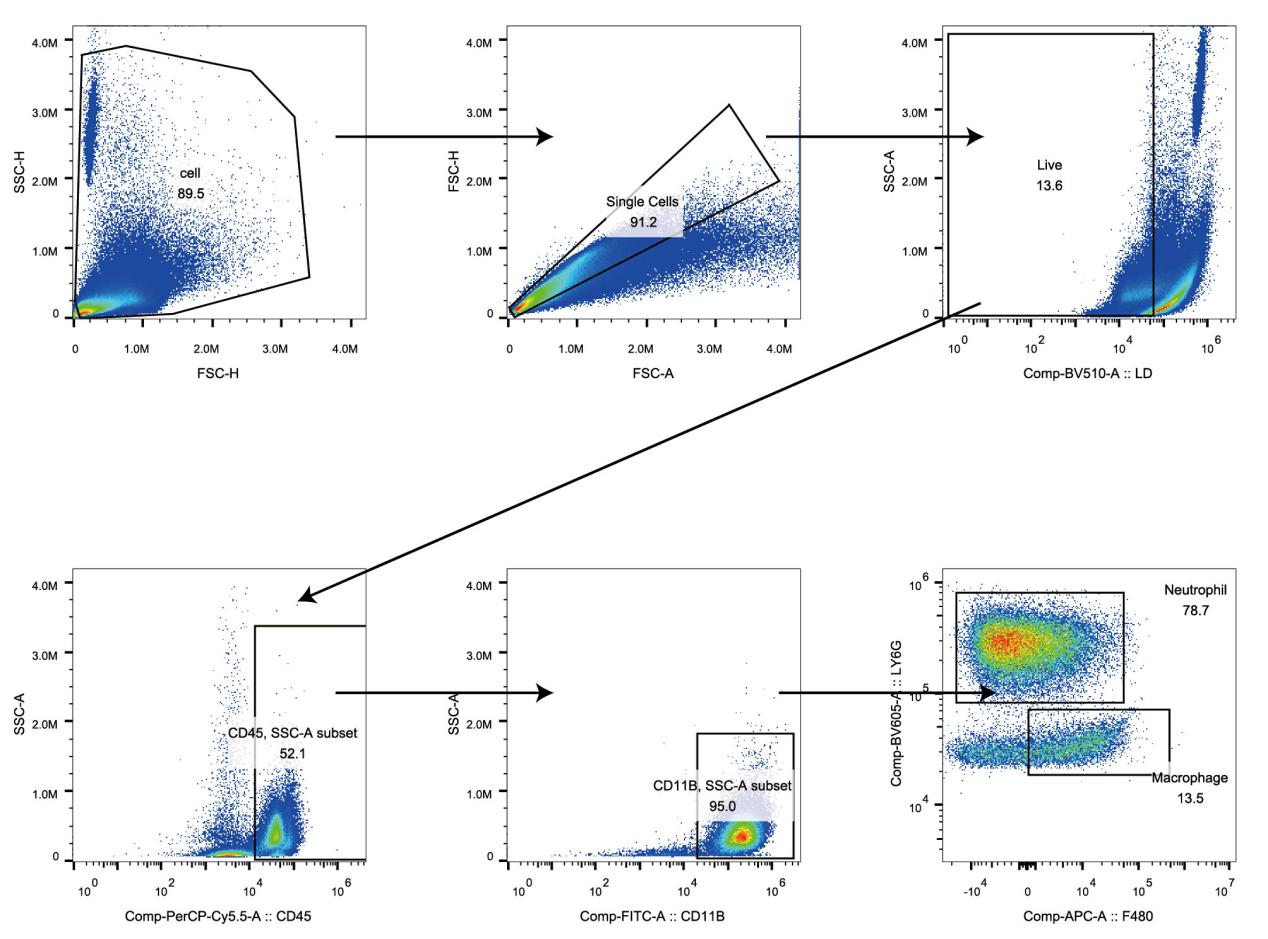


**Figure S15.** The gating strategy for the leukocytes in the heart.


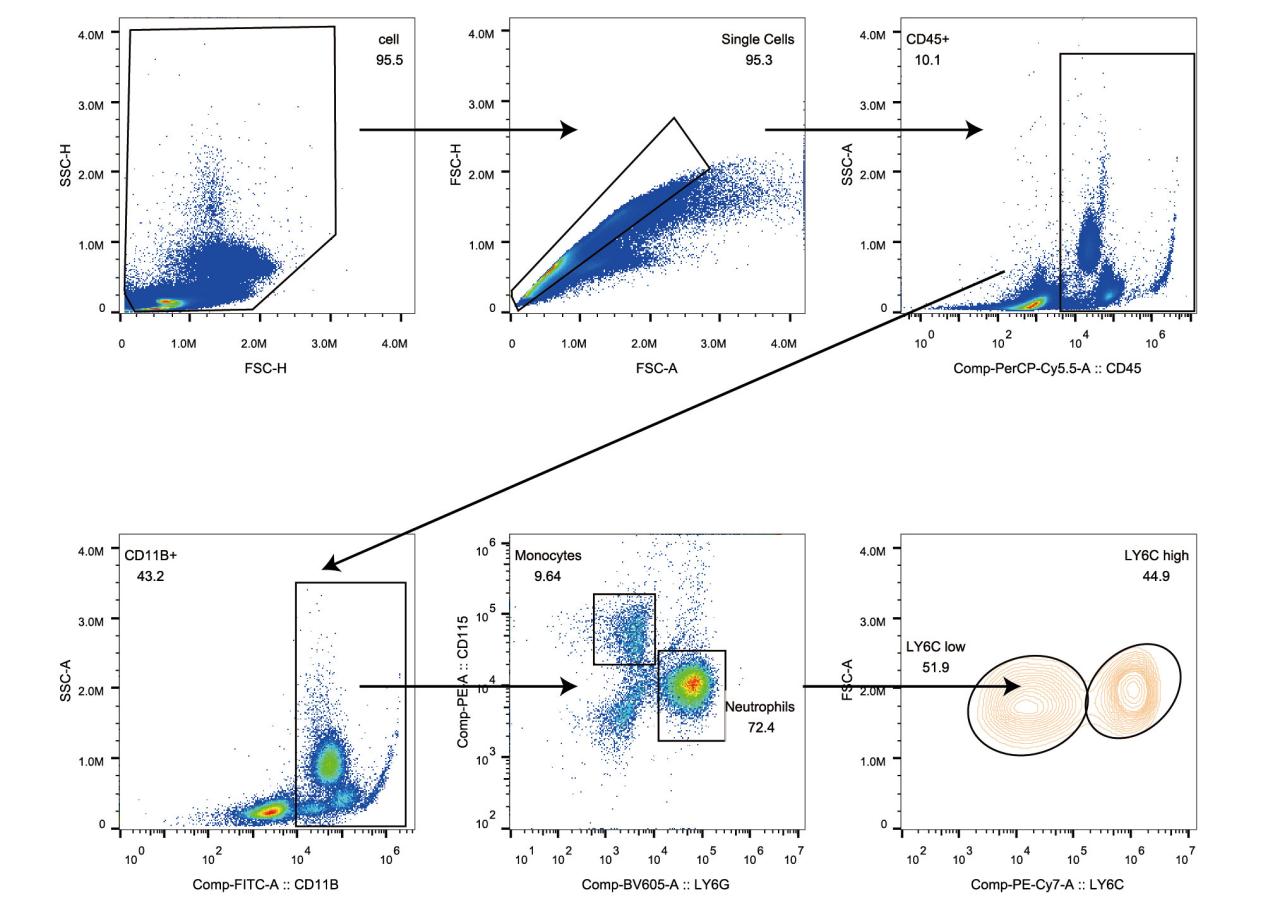


**Figure S16.** The gating strategy for peripheral blood leukocytes.


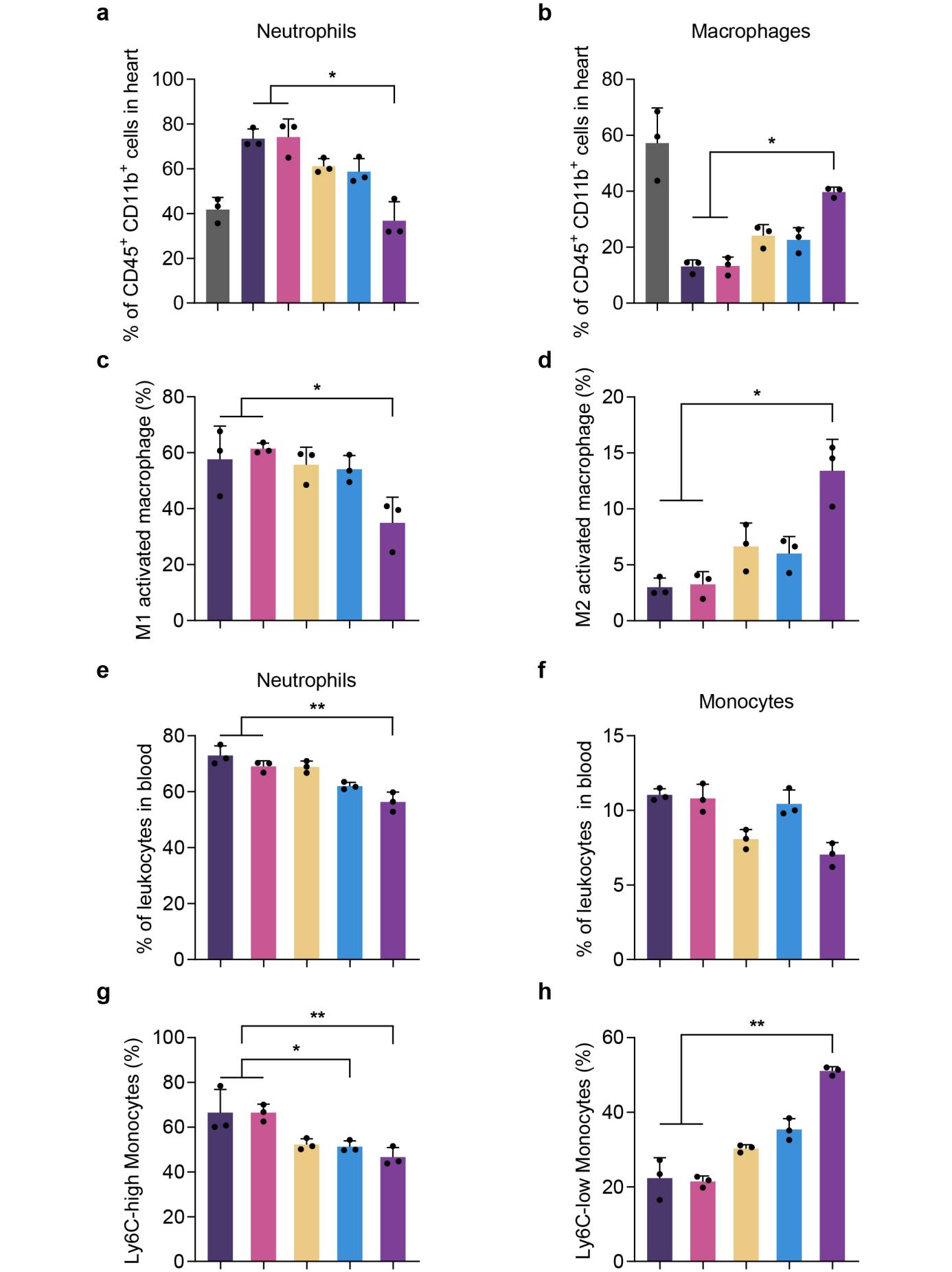


**Figure S17.** The quantitative analysis of the percantages of neutrophils (a), macrophages (b), M1 activated macrophages (c), M2 activated macrophage (d) in the heart, and the proportions of leukocytes (e), monocytes (f), Ly6C-high monocytes (g) and Ly6c-low monocytes (h) in the blood from Figure 4**.**


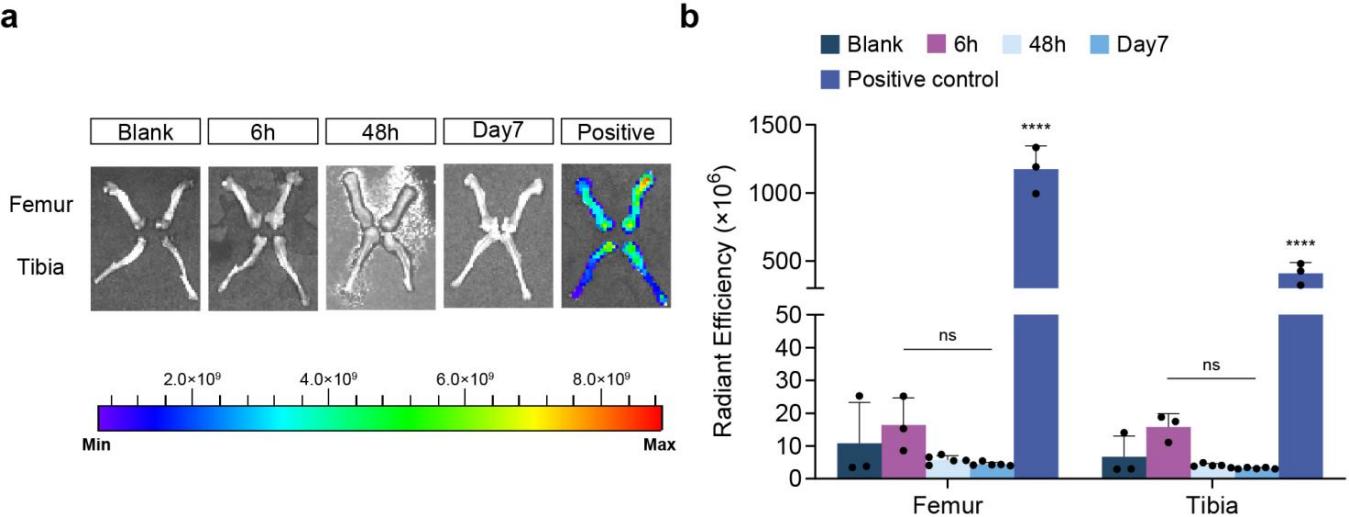


**Figure S18. NE-ABR-Cu_x_O nanocomplexes did not distribute in the bone marrow after intraperitoneal injection.** (a) Representative *ex vivo* fluorescent images and (b) quantitative analysis (n = 3-5) of lower limb bones (femur and tibia) derived from C57BL/6 male mice intraperitoneally injected with NE-ABR-Cu_x_O after 6 h, 48 h and 7 days. In the positive control group, equal amount of NE-ABR-Cu_x_O-Cy5.5 was directly injected with into the bone marrow cavity.

**
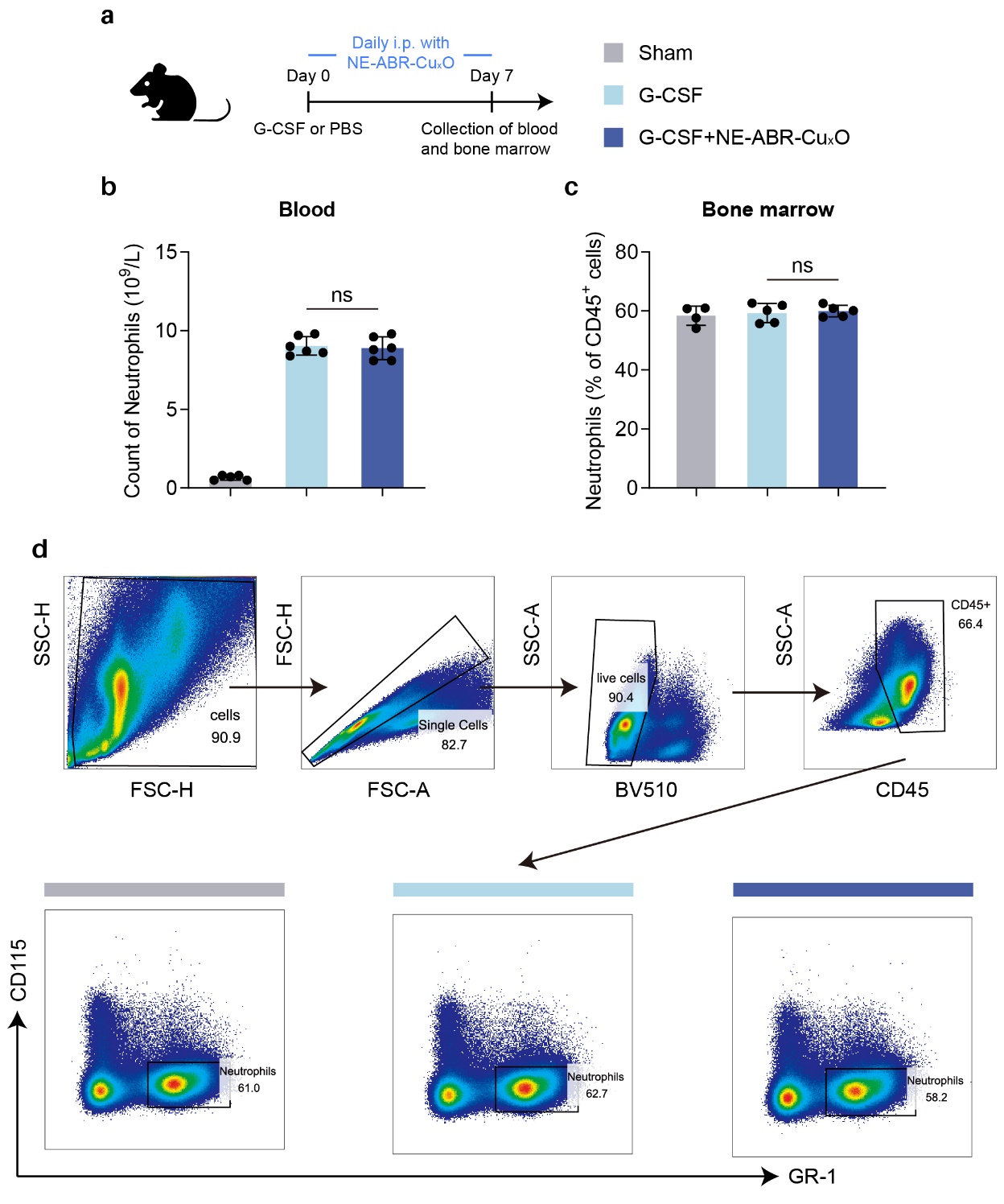
**

**Figure S19.** (a) A scheme showing the experimental schedule and treatments. (b) Counts of neutrophils derived from routine blood tests, and (c) flow cytometry analysis showig the proportions of neutrophils in CD45^+^ cells in bone marrow derived from mice with different treatments as indicated. (d) The gating strategy for neutrophils in bone marrow.


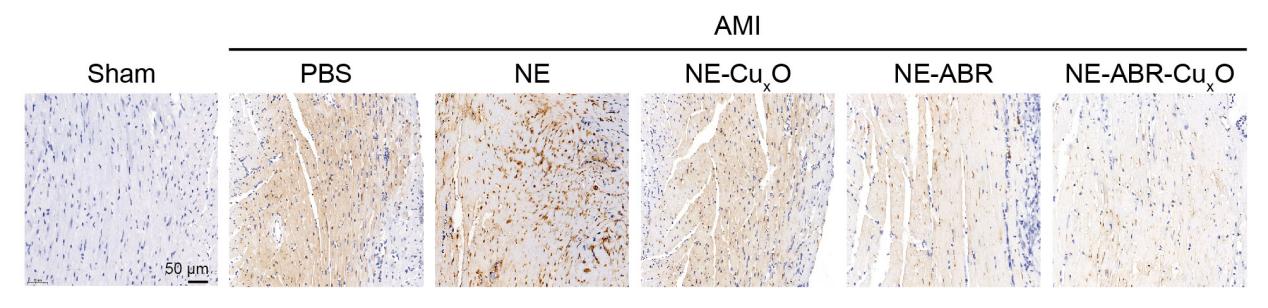


**Figure S20.** AMI-induced apoptosis was significantly ameliorated in the NE-ABR-, NE-Cu_x_O- and NE-ABR-Cu_x_O-treated groups.


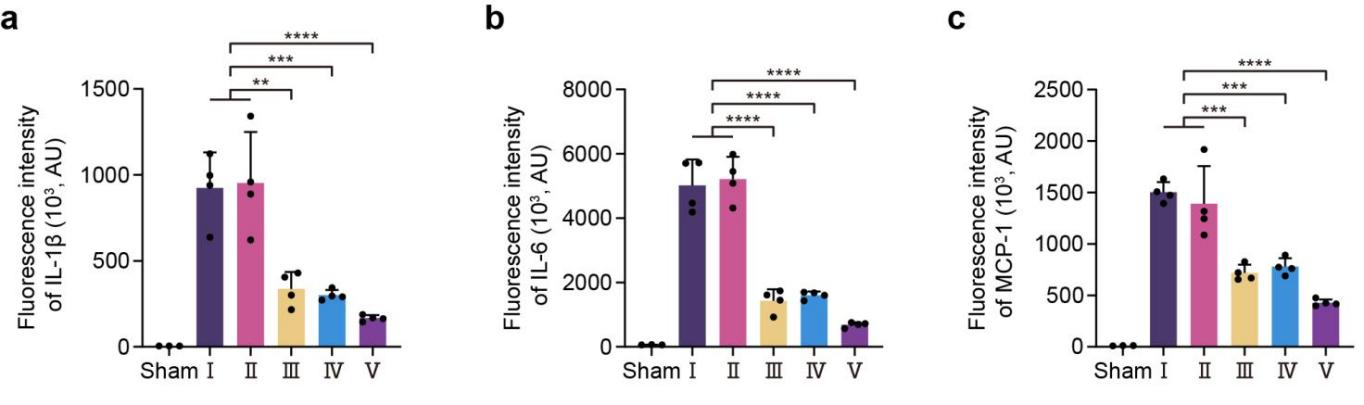


**Figure S21.** The quantitative analyses of fluorescence intensity of (a) IL-1β, (b) IL-6 and (c) MCP-1 from Figure 5.
